# Structure Guided Discovery of Ancestral CRISPR-Cas13 Ribonucleases

**DOI:** 10.1126/science.adq0553

**Published:** 2024-07-18

**Authors:** Peter H. Yoon, Zeyuan Zhang, Kenneth J. Loi, Benjamin A. Adler, Arushi Lahiri, Kamakshi Vohra, Honglue Shi, Daniel Bellieny Rabelo, Marena Trinidad, Ron S. Boger, Muntathar J. Al-Shimary, Jennifer A. Doudna

**Affiliations:** 1 Department of Molecular and Cell Biology, University of California, Berkeley; Berkeley, CA, USA.; 2 Innovative Genomics Institute; University of California, Berkeley, CA, USA.; 3 Howard Hughes Medical Institute, University of California, Berkeley; Berkeley CA, USA.; 4 Biophysics Graduate Group, University of California, Berkeley; Berkeley, CA, USA.; 5 California Institute for Quantitative Biosciences, University of California, Berkeley; Berkeley, CA, USA.; 6 Gladstone Institutes; San Francisco, CA, USA.; 7 Gladstone-UCSF Institute of Genomic Immunology; San Francisco, CA, USA.; 8 Molecular Biophysics and Integrated Bioimaging Division, Lawrence Berkeley National Laboratory; Berkeley, CA, USA.; 9 Department of Chemistry, University of California, Berkeley; Berkeley, CA, USA.

## Abstract

The RNA-guided ribonuclease CRISPR-Cas13 enables adaptive immunity in bacteria and programmable RNA manipulation in heterologous systems. Cas13s share limited sequence similarity, hindering discovery of related or ancestral systems. To address this, we developed an automated structural-search pipeline to identify an ancestral clade of Cas13 (Cas13an), and further trace Cas13 origins to defense-associated ribonucleases. Despite being one third the size of other Cas13s, Cas13an mediates robust programmable RNA depletion and defense against diverse bacteriophages. However, unlike its larger counterparts, Cas13an uses a single active site for both CRISPR RNA processing and RNA-guided cleavage, revealing the ancestral nuclease domain has two modes of activity. Discovery of Cas13an deepens our understanding of CRISPR-Cas evolution and expands opportunities for precision RNA editing, showcasing the promise of structure-guided genome mining.

Type VI CRISPR-Cas systems provide adaptive immunity in prokaryotes by targeting RNA transcripts of invading mobile genetic elements (1–3). Interference is mediated by the Cas13 protein and its CRISPR RNA (crRNA) that together form an RNA-guided ribonuclease, whose simple reprogrammability has facilitated widespread repurposing in biotechnology (3–9). The defining feature of Cas13 is a pair of higher eukaryotes and prokaryotes nucleotide-binding (HEPN) domains. In Cas13, the two HEPN domains (HEPN1 and HEPN2) dimerize intramolecularly to form the active site in response to target-transcript recognition, which contrasts other HEPN proteins that typically homodimerize (10, 11). The HEPN superfamily of ribonucleases exhibits great sequence and structural diversity (10, 11). Even within the Cas13 family, the lack of sequence conservation makes conventional homology searches and evolutionary analyses difficult. As a result, compared to other Class 2 CRISPR-Cas effectors including Cas9 (Type II) and Cas12 (Type V), few distinct Cas13 subtypes have been identified to date, and little is known about their evolutionary origins.

Structural comparisons offer a solution to the challenges posed by low sequence conservation within protein families like Cas13, as protein folds exhibit greater conservation (12). Historically, the limited availability of protein structures has bottlenecked structure-centric evolutionary analyses. However, the advent of atomic-accuracy prediction programs and their associated databases, which now approach a billion structures, has largely overcome this obstacle (13–15). Nevertheless, exploiting these databases poses new challenges, as traditional structural comparison programs were not designed for such scale. To address this, machine learning programs such as Foldseek (16) accelerate structural homology searches compared to gold standard programs like DALI or TMalign (17, 18). However, machine learning-based programs are less sensitive than traditional programs (16, 19), highlighting the need for scalable approaches with maximal sensitivity to uncover novel relationships.

## Automated structural homology search uncovers ancient CRISPR-Cas13 systems

**Fig. 1:**
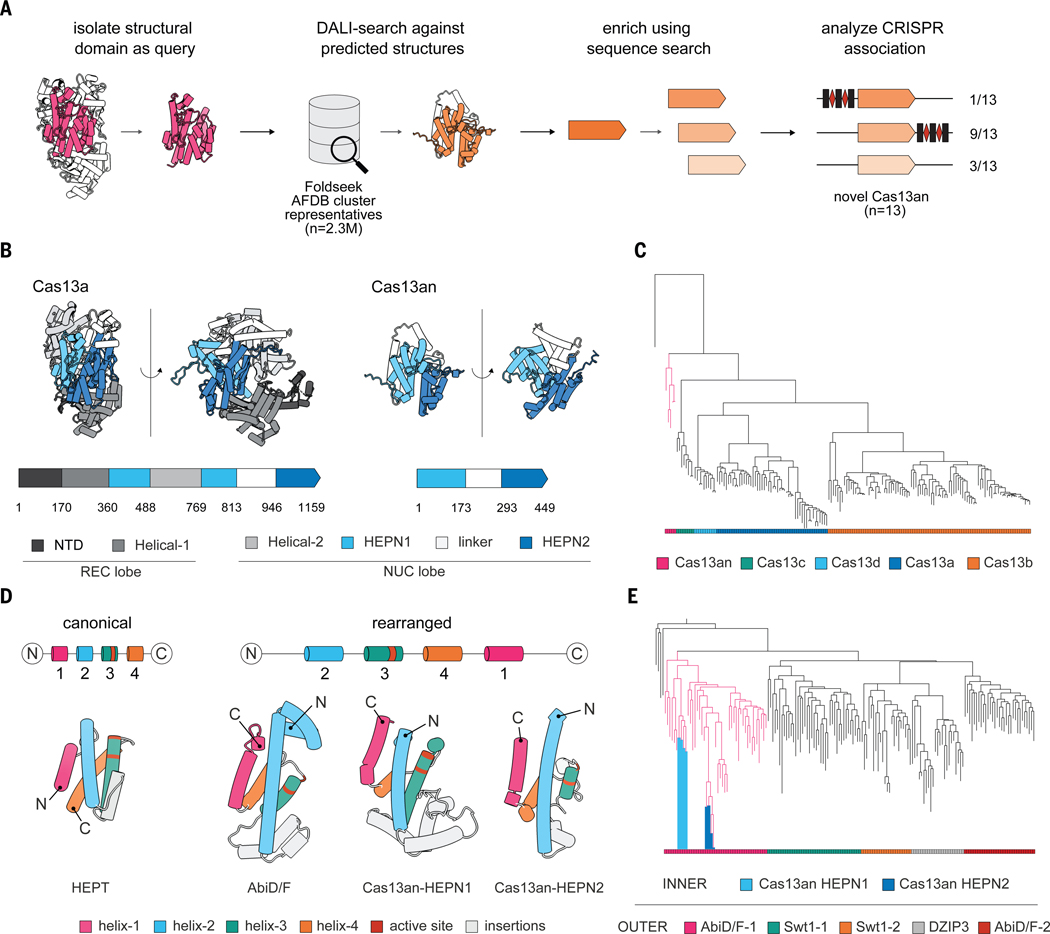
Structural homology search enabled discovery of ancestral Cas13 systems. (**A**) Schematic of automated structure-based discovery pipeline. AFDB, AlphaFold Database. (**B**) Comparison of Cas13a (PDBID: 5XWY) and Cas13an (AFDBID: A0A7C5SD50) structure and domain architecture. (**C**) Maximum-likelihood phylogenetic tree of Cas13 subtypes. Sequences are provided in table S6, and alignment and tree files are provided in data S1. (**D**) Structural comparison of HEPN domains with canonical organization (HEPT; PDBID: 5YEP) and shared rearrangements (Cas13an HEPN1 and HEPN2 domains; AFDBID: A0A7C5SD50, and AbiD/F gene; AFDBID: A0A3S5XYX8). (**E**) Maximum-likelihood phylogenetic tree of Cas13an HEPN1 and HEPN2 domains and their structural homologs. Sequences and annotations of these proteins are available in table S9, and alignment and tree files are provided in data S1.

Motivated by this challenge, we developed an automated structural-search pipeline that combines the speed of machine learning-based search methods with the sensitivity of traditional structure alignment programs. Specifically, we leveraged a Foldseek-clustered AlphaFold database (20), whose reduced search space makes slow-but-sensitive DALI-searches feasible (Methods). Using representative HEPN dimers within known Cas13 proteins (21–23) as the search query (Fig. 1A), we found twelve previously uncharacterized protein clusters in the AlphaFold database bearing an intramolecular HEPN dimer (fig. S1 and table S1, 2). Further sequence-based homology searches and genomic analyses revealed that two of the newly identified clusters occur next to CRISPR arrays, representing a new Cas13 subtype (Cas13an) (Fig. 1A and fig. S2). Notably, neither Foldseek nor hidden Markov model searches were able to detect significant homology between previously known Cas13s and Cas13an (fig. S3). This highlights the considerable divergence of Cas13an compared to known Cas13 proteins, and underscores the importance of sensitive search strategies.

In total, we identified thirteen diverse Cas13an sequences, ten of which occur next to CRISPR arrays with conserved repeat sequences (fig. S2, 4 and table S3). Notably, all Cas13an loci lacked other Cas genes, including the acquisition-associated genes *cas1* and *cas2* (fig. S2). Nevertheless, the CRISPR arrays appeared to be actively acquiring new spacers, which we found target double-stranded DNA (dsDNA) phages (fig. S5 and table S4, 5). Consistent with this, four of the ten Cas13an encoding genomes have *cas1* and *cas2* genes in *trans* belonging to Type II CRISPR-Cas systems (fig. S6). This raises the possibility that Cas13an hijacks adaptation modules from Type II systems, as previously suggested for other CRISPR-Cas systems (24, 25).

Ranging in size from 429 to 577 amino acids, Cas13an proteins are remarkably small compared to previously characterized Cas13 orthologs, which are typically 800–1400 amino acids in length (1, 26). Structural comparisons suggest that Cas13an’s compact size is due to a lack of large insertions in the HEPN domains and the absence of a canonical REC lobe that functions to bind the crRNA in Class 2 CRISPR-Cas effectors (Fig. 1B). The underdeveloped REC lobe in Cas13an is reminiscent of diminutive REC lobes in smaller Cas9s and Cas12s and their ancestral proteins, IscB and TnpB (27, 28), suggesting that Cas13an may represent an early evolutionary form of Cas13.

Motivated by this unusual predicted structure, we next explored the evolutionary relationship of Cas13an to other Cas13 subtypes. Combining structural and sequence-based phylogenetic analyses (Methods) revealed that Cas13an is likely ancestral to other proteins in the lineage (Fig. 1C, table S6 and data S1). This suggests that all three Class 2 CRISPR-Cas effectors—Cas9, Cas12, and Cas13—originated from compact ancestral proteins that underwent domain accretion over time. We also noticed that within the compact architecture of Cas13an, the primary sequences of the two HEPN domains were similarly rearranged compared to canonical HEPN proteins, implying a close relationship (Fig. 1D). To test this hypothesis, we isolated Cas13an HEPN1 and HEPN2 domains as separate queries for our structural-search pipeline. Across both searches, the shared hits revealed that Cas13an HEPN domains bear structural similarities to the non-CRISPR HEPN nucleases Swt1, DZIP3 and AbiD/F (table S7, 8). Notably, phylogenetic analysis revealed that both Cas13an HEPN domains form a clade with AbiD/F (Fig. 1E, table S9 and data S1), which are phage defense-associated ribonucleases predicted to co-occur with a non-coding RNA of unknown function (RNA family: RF03085) (29). Summarizing our findings, we propose that Cas13 evolved from compact ancestral enzymes formed by the fusion of two closely related defense-associated HEPN genes, with potential RNA-guided capabilities predating the Cas13 lineage.

## Compact Cas13s mediate potent programmable RNA interference *in vivo*

**Fig. 2.**
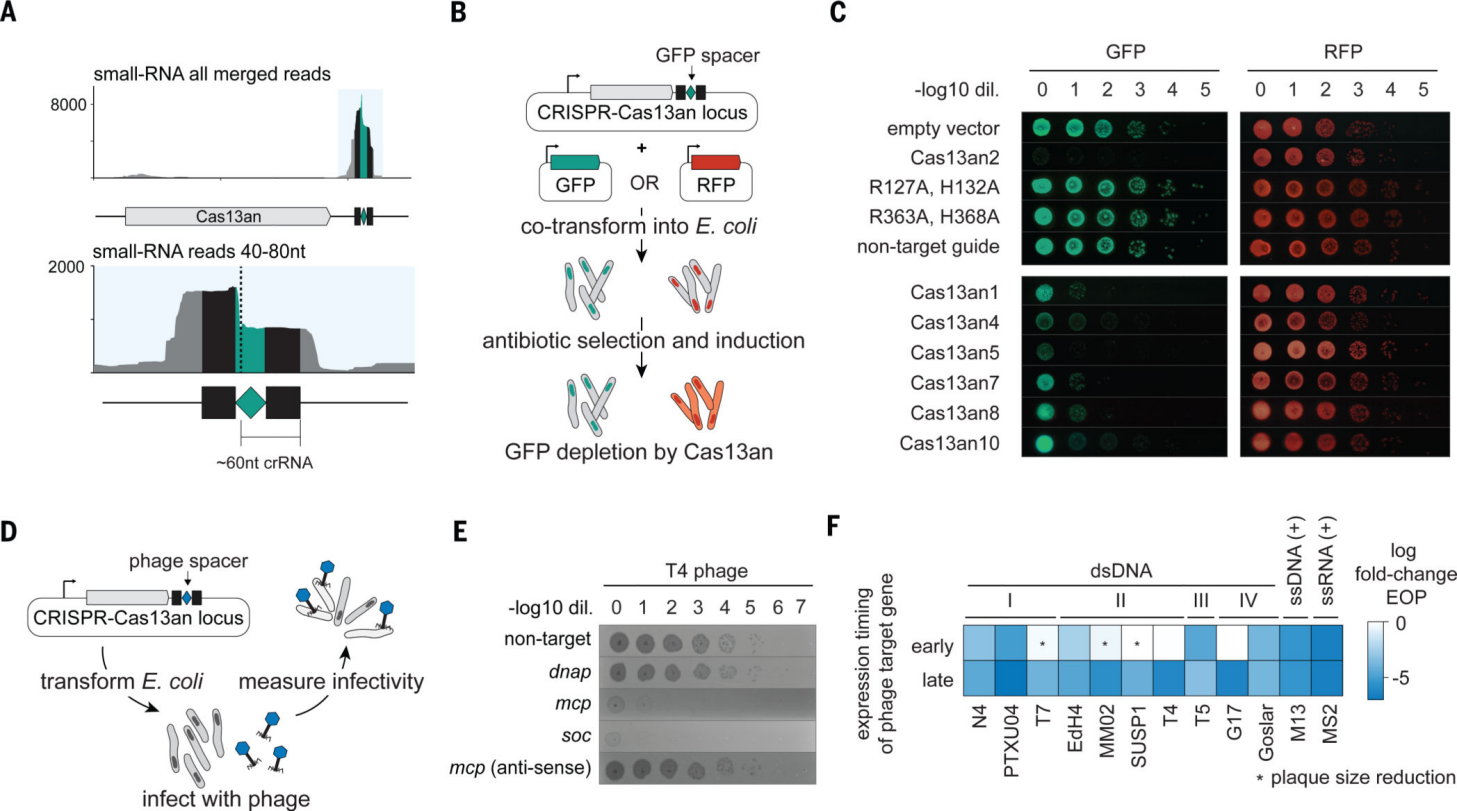
Cas13an systems provide targeted RNA knockdown and defense against phages. (**A**) Small RNA-sequencing of CRISPR-Cas13an1 locus heterologously expressed in *E. coli*. Inset shows reads of length 40–80 nucleotides (nt) corresponding to processed crRNA. Black squares denote CRISPR-repeat, and green diamond denotes spacer sequence. (**B**) Schematic of green-fluorescent protein (GFP) depletion assay in *E. coli*. (**C**) Serial dilutions of *E. coli* in GFP depletion assays. Each spot progression represents a 10-fold dilution. (**D**) Schematic of phage challenge assays in *E. coli*. (**E**) Phage challenge assay results for lytic T4-phage using Cas13an8. Each spot progression represents a 10-fold dilution of phage stock. (**F**) Efficiency of plaquing (EOP) summary of Cas13an8 targeting phages of unrelated, diverse genera and labeled by genome nucleic acid composition. Labels I, II, III, and IV represent Podovirus, Myovirus, Siphovirus, and Jumbo Myoviruses respectively.

Building on our bioinformatics insights, we next examined CRISPR-Cas13an systems using *E. coli* as a heterologous host to determine their active components. To test the hypothesis that the Cas13an-adjacent CRISPR array encodes guide RNAs, we transformed *E. coli* with plasmids encoding a Cas13an, a CRISPR array, and intergenic regions between the two. Small RNA-sequencing revealed the expression of crRNAs whose spacer sequence is positioned before the repeat region (Fig. 2A and fig. S7). This distinctive arrangement is observed only in crRNAs of the Cas13b family, which includes variants previously reported as Cas13X, Cas13Y, and Cas13e–i (30, 31). In light of Cas13an, this unusual RNA arrangement can be reinterpreted to be an ancestral characteristic instead of a derived one.

Next, we tested the possible crRNA-guided ribonuclease activity of Cas13an by targeting transcripts of the green fluorescent protein (GFP). We co-transformed *E. coli* with plasmids encoding either GFP or the related red fluorescent protein (RFP) along with separate plasmids encoding various Cas13an orthologs with GFP-targeting crRNAs (Fig. 2B and table S10). We observed diminished GFP but not RFP fluorescence, consistent with Cas13an’s specificity as an RNA-guided nuclease (Fig. 2C and fig. S8). GFP expression was unaffected in this experiment when a non-targeting crRNA was used (Fig. 2C). Furthermore, point mutations in the active site of either HEPN domain abolished Cas13an-mediated GFP reduction, confirming the critical role of the HEPN domains in this activity (Fig. 2C). These results show that the Cas13an system is the most compact CRISPR-Cas effector complex known to date, comprising a protein as small as 429 amino acids and a single RNA component of ~60 nucleotides (nt) in length.

Motivated by these results and the previously established efficacy of larger Cas13 systems in antiphage defense (6), we investigated whether Cas13an could similarly protect against phage infection. To do this, we challenged *E. coli* harboring plasmids encoding Cas13an8, which was chosen for its robust activity, using the lytic dsDNA phage T4 (Fig. 2D). We found that when expressed together with a T4-phage-targeting crRNA (table S10), Cas13an provided >10^3^-fold protection, as shown by phage plaquing efficiencies. Notably, this effect was independent of the essentiality of the targeted phage gene, as targeting the non-essential *soc* gene restricted phage replication (Fig. 2E). This implies Cas13an provides phage-defense through both targeted RNA cleavage in *cis*, and indiscriminate cleavage in *trans* like other Cas13 subtypes (2, 3, 7, 32, 33). crRNAs matching the antisense (non-coding) phage DNA strand had no effect on phage plaquing, consistent with Cas13an’s targeting of RNA (Fig. 2E). We next tested whether robust defense observed for T4-phage extends to a broader spectrum of phages of different genera and life cycles (6). We found all phages tested to be susceptible to at least one phage-targeting crRNA (Fig. 2F, fig. S9 and table S10). Similar to what was reported for Cas13a, targeting late rather than early genes was reliably more effective (6, 33). We conclude that despite its small size, Cas13an provides potent, broad-spectrum defense against diverse phages, suggesting this trait is ancestral in the Cas13 lineage.

## Ancestral HEPN domain active site is a multifunctional ribonucleolytic center

**Fig. 3.**
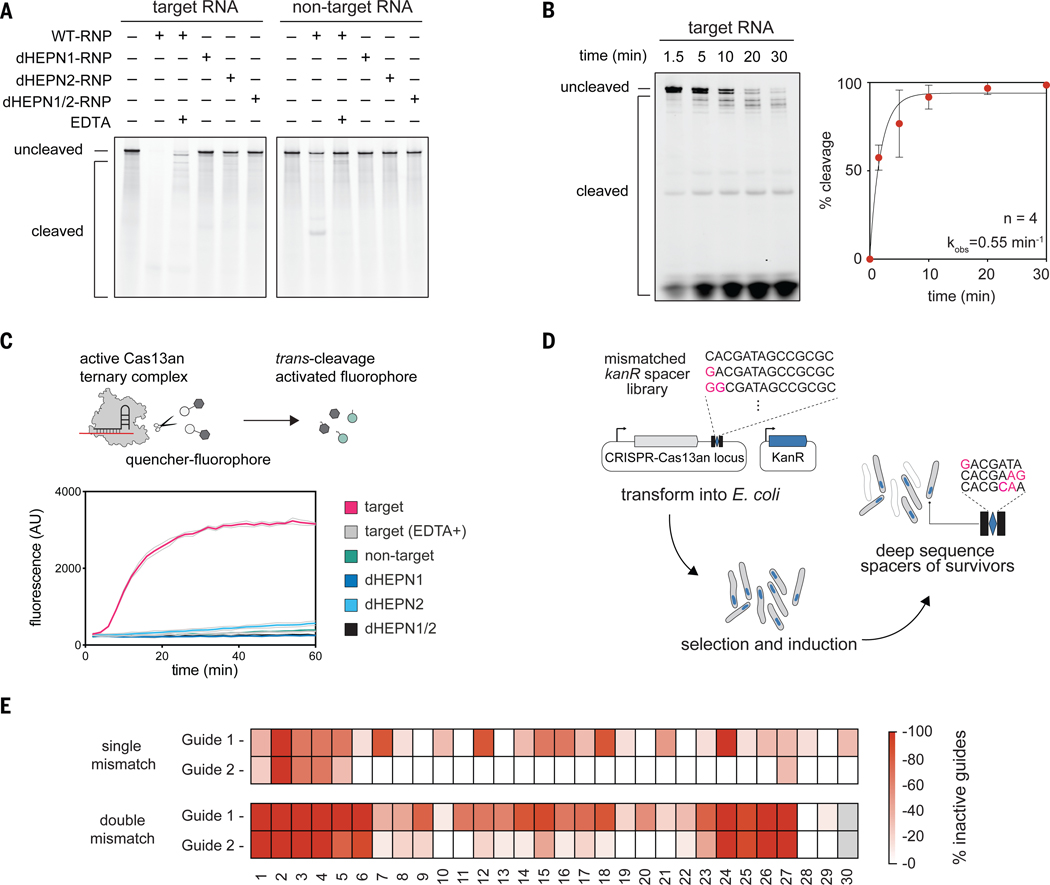
Complementary RNA triggers both *cis*- and *trans*-cleavage in Cas13an. **(A)** End point measurement (60 min) of Cas13an2 ribonucleoprotein complex (RNP) cleavage of 5’-fluorescein (FAM)-labeled guide complementary (target RNA) and non-complementary (non-target RNA) substrates. In mutants, alanine substitutions were introduced in HEPN Rx4H motifs: R127A/H132A for dHEPN1, R363A/H368A for dHEPN2, and all four for dHEPN1/2. **(B)** Kinetics of Cas13an2 RNP mediated target RNA cleavage (*cis*-cleavage). **(C)** Schematic of fluorophore-quencher assay to measure collateral RNA cleavage (*trans*-cleavage) induced by Cas13an2 target RNA recognition (top). *Trans*-cleavage induced fluorescence traces for Cas13an RNP with target RNA and controls (bottom). AU, arbitrary units. **(D)** Schematic of mismatch tolerance assay in *E. coli*. **(E)** Heatmap representation of Cas13an2 mismatch tolerance for single and double mismatch spacers.

Having established the *in vivo* activity of Cas13an, we chose Cas13an2 for biochemical analysis due to its *in vitro* stability. Using purified Cas13an protein produced in *E. coli*, together with *in vitro* transcribed full-length crRNAs, we observed that Cas13an cleaved RNAs bearing sequence complementarity to the 30nt crRNA spacer sequence (Fig. 3A, B and table S11). Additionally, Cas13an exhibited *trans*-cleavage activity, indiscriminately cleaving a fluorophore-quencher-labeled reporter RNA in the presence of a crRNA-complementary RNA target (Fig. 3C). Though direct comparisons are difficult, the *trans*-cleavage rate for Cas13an appeared to be slower than those of previously reported Cas13 variants (3, 21, 34, 35), and was instead more comparable to ssDNA *trans*-cleavage rates of Cas12 proteins (36, 37). In control experiments, we observed cleavage inhibition in Cas13an proteins bearing alanine substitutions in the conserved Rx4H motifs of the HEPN domain active sites (Fig. 3A, C). We also observed inhibition of Cas13an activity in presence of the Mg^2+^ chelator EDTA (Fig. 3A, C). However, this inhibitory effect was less pronounced than that observed for other Cas13 subtypes (2, 3, 7, 21), as EDTA addition did not completely abolish *cis*-cleavage (Fig. 3A).

To further investigate target recognition requirements for Cas13an, we designed a comprehensive mismatch library of spacers with disrupted complementarity to the kanamycin resistance (*kanR*) gene in *E. coli* (Fig. 3D and table S12). We co-transformed *E. coli* with helper plasmids encoding Cas13an2 systems alongside a separate target plasmid containing the *kanR* gene. Cultivation on kanamycin-containing plates resulted in the selective survival of cells with defective spacers. Sequencing of surviving cells showed that tolerance for single-base mismatches was target sequence dependent (Fig. 3E). In contrast, tolerance for double mismatches appeared consistent between targets, with complementarity in the 1–6nt regions and 24–27nt regions of the spacer being crucial for Cas13an activity *in vivo* (Fig. 3E). We next modified the mismatch assay to test whether sequences flanking the target site influence Cas13an cleavage efficacy (fig. S10, Methods). This revealed that Cas13an2 did not have a strong preference for nearby bases, suggesting a flexible target scope (fig. S10).

**Fig. 4.**
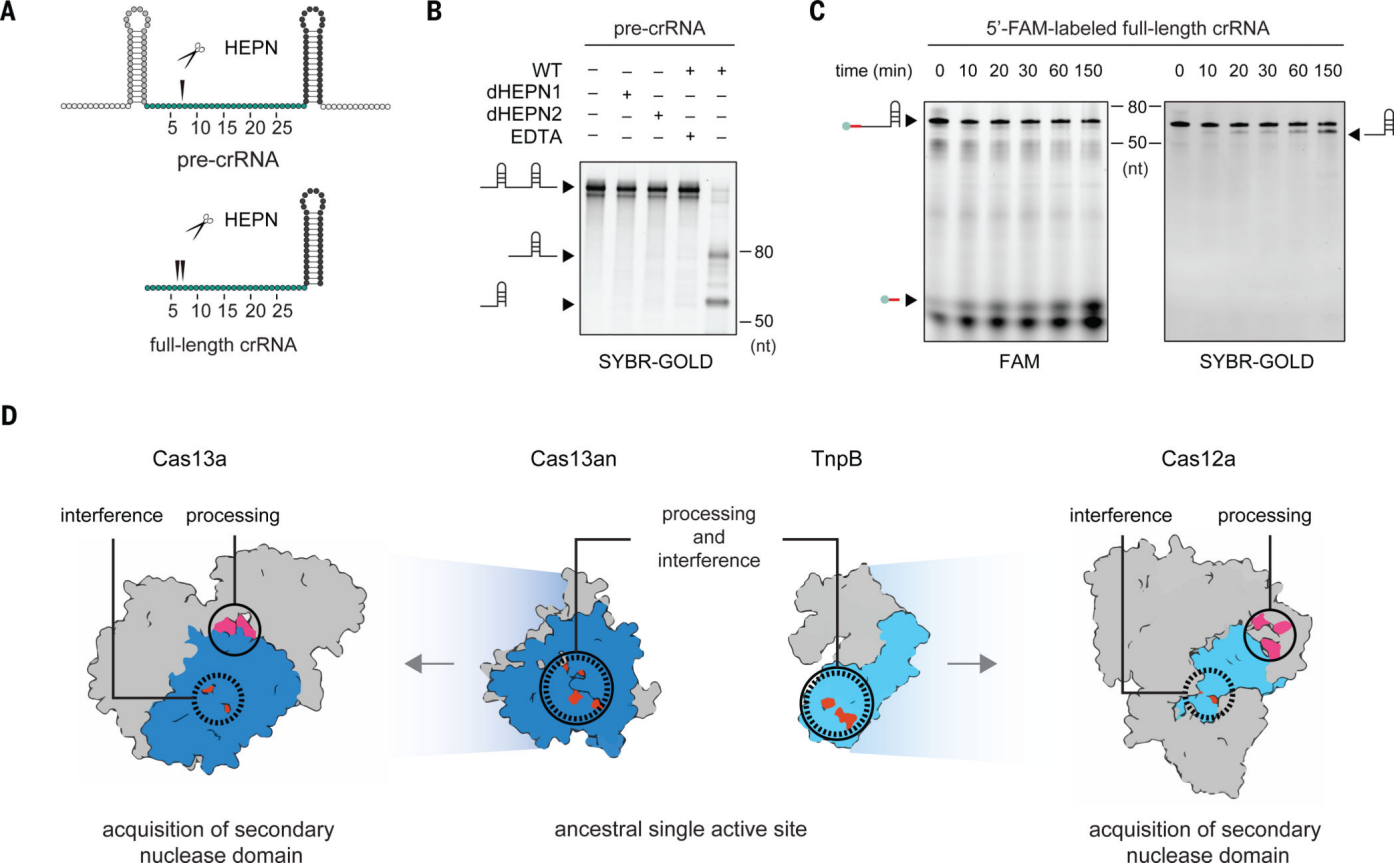
Multifunctional HEPN domains enable RNA-guided cleavage and pre-crRNA processing in Cas13an. **(A)** Substrates used for RNA processing assays. Top: pre-crRNA and processing site (black triangle) inferred from RNA-sequencing (fig. S12). Bottom: full-length crRNA and processing sites inferred from denaturing gels (fig. S11C). **(B)** *In vitro* pre-crRNA processing by Cas13an2. Cas13an processes pre-crRNA in vitro only in the presence of Mg2+ and catalytic residues of both HEPN domains are necessary for processing. Gel with the ladder is attached in fig. S11A. **(C)** *In vitro* RNA processing of 5’-FAM-labeled full-length crRNA. Same gel was first examined for FAM signal, and then for SYBR-GOLD stain signal. Gel with the ladder attached is shown in fig. S11B. **(D)** Parallels in evolutionary paths between two unrelated Class 2 CRISPR-Cas effectors, Cas13 and Cas12.

In addition to cleaving transcripts in response to target RNA recognition, previously characterized Cas13 subtypes mediate processing of CRISPR array derived pre-crRNA into individual crRNAs (3, 7, 32). crRNA maturation enhances interference and is a crucial regulatory step conserved across CRISPR-Cas systems. In previously studied Cas13 subtypes, maturation is catalyzed by a Mg^2+-^independent active site distinct from the Mg^2+-^dependent HEPN active site used for targeted RNA cleavage (3, 21, 38). The minimal architecture of Cas13an and absence of a recognizable pre-crRNA processing center made it unclear whether Cas13an could also mediate maturation. To test this, we analyzed Cas13an-mediated cleavage of *in vitro* transcribed pre-crRNA containing a repeat-spacer-repeat sequence flanked by additional 15nt (Fig. 4A and table S11). Denaturing gel analysis revealed Cas13an-dependent pre-crRNA processing, in which site-specific RNA cleavage occurred only within the spacer sequence (Fig. 4A, B and fig. S11A), further confirmed by RNA-sequencing data for the reaction (fig. S12). To unambiguously determine the orientation of cleavage, we repeated the experiment with a 5’ labeled crRNA containing the full-length 30nt spacer followed by a 36nt complete repeat (Fig. 4A and table S11). This resulted in a 6–7nt labeled product, revealing that Cas13an cleaved 24 or 23nt upstream of the repeat (Fig. 4A, C and fig. S11B, C), consistent with the *in vivo* small RNA-sequencing results (Fig. 2A and fig. S7). *In vitro* cleavage using crRNAs with shorter spacers showed that the optimal spacer length is 30nt (fig. S13 and table S11), in agreement with the observed importance of the 1–6nt region for *in vivo* interference (Fig. 3E). The discrepancy between the spacer length of mature crRNA (23–24nt) and optimal crRNA (30nt) warrants future investigation of the interplay between processing and interference.

The unique pre-crRNA cleavage pattern of Cas13an suggests a processing mechanism distinct from other Cas13 subtypes, whose pre-crRNA cleavage occurs near the repeat sequence (fig. S14). Experiments showed that adding EDTA inhibited Cas13an-mediated pre-crRNA maturation, indicating Mg^2+^ dependence of the reaction (Fig. 4B). We hypothesized that the Mg^2+-^dependent HEPN domains are responsible for crRNA maturation, similar to the multifunctional nucleic acid processing domains found in compact Cas12 enzymes and their TnpB ancestors (fig. S15) (39, 40). Notably, neither addition of EDTA nor mutations in the HEPN domains affected binding of Cas13an to crRNA, based on electrophoretic mobility shift assays (EMSAs) (fig. S16). However, HEPN domain mutations ablated Cas13an-catalyzed pre-crRNA processing, uncovering a dual functionality of the HEPN domains that enables both target RNA cleavage and guide RNA processing (Fig. 4B).

These findings reveal an unanticipated convergence among ancestral Class 2 CRISPR-Cas nucleases to license a single active site for cleavage of both target nucleic acids and guide RNA (Fig. 4D and fig. S15). Potentially, other HEPN nucleases structurally similar to Cas13 identified in this study also possess such dual functionality (Fig. 1D, E, fig. S1 and table S1, 2, 7). The parallels observed between the evolution of RNA-targeting Cas13 and DNA-targeting Cas12 highlight recurrent solutions to address shared evolutionary pressures imposed on small RNA-guided nucleases (Fig. 4D and fig. S14, 15). This parallel is further reinforced by secondary pre-crRNA processing active sites having been acquired on multiple independent occasions within both the Cas13 and Cas12 lineages (fig. S14, 15). Collectively, these insights not only bridge a significant gap in our understanding of Class 2 CRISPR-Cas system origins but also establish a foundation for future exploration of the evolution and mechanisms of RNA-guided ribonuclease activity.

In this study, we developed and applied a structure-based search strategy that combines rapid clustering and sensitive comparisons to uncover homology between highly divergent proteins. We further leveraged structural and sequence comparisons to resolve complex phylogenetic relationships, enabling the discovery of recurrent themes underlying CRISPR-Cas enzyme evolution. Although this study focused on the AlphaFold database, our strategy generalizes to other structure prediction databases, including those of metagenomic or viral origin (15, 41). As structure prediction methods and associated databases continue to advance, structure-guided protein mining will become increasingly powerful, enabling greater access to biological insights that have long evaded detection. This study paves the way for future investigations of shared folds and functions across remote homologs, which will further illuminate principles underlying biomolecular evolution.

## Supplementary Material

Yoon et al Supplementary Material

Yoon et al Supplementary Data 1-3

Yoon et al Supplementary Tables 1-12
