# Supplementary material for: Structure Guided Discovery of Ancestral CRISPR-Cas13 Ribonucleases
Source: Science. Author manuscript; Available in PMC 2025 Jun 13. (PMC12165695; doi:10.1126/science.adq0553)
Supplement: Yoon et al Supplementary Material [file NIHMS2074710-supplement-Yoon_et_al_Supplementary_Material.docx]

Supplementary Materials for

**Structure Guided Discovery of Ancestral CRISPR-Cas13 Ribonucleases**

**Authors:** Peter H. Yoon^1,2,3^, Zeyuan Zhang^2,3,4,5^, Kenneth J. Loi^1,2^, Benjamin A. Adler^2,3,5^, Arushi Lahiri^1,2^, Kamakshi Vohra^2,3^, Honglue Shi^2,3^, Daniel Bellieny Rabelo^2,5^, Marena Trinidad^2,3^, Ron S. Boger^2,3,4^, Muntathar J. Al-Shimary^1,2,3^ and Jennifer A. Doudna^1,2,3,5,6,7,8,9^*

*Corresponding author. Email: doudna@berkeley.edu

**The PDF file includes:**

Materials and Methods

Figs. S1 to S16

References (43-54)

**Materials and Methods**

Structural-search and novel CRISPR system identification pipeline

Manual examination of structures of previously characterized Cas13 subtypes led to the curation of the conserved intramolecular HEPN dimer core. For Cas13a, Cas13b, and Cas13d, experimental structures were used (PDBID: 5XWP, 6DTD, 6E9F, respectively). For Cas13c, which lacks an experimental structure, a ColabFold [(*43*)](https://www.zotero.org/google-docs/?xKSYZn) model of A0A9X2MGT7 was generated using three recycles without amber relaxation. The HEPN core regions were isolated from the Cas13 protein structures using ChimeraX [(*44*)](https://www.zotero.org/google-docs/?zGvA0R) and used as queries for the structural-search. DALI (DaliLite.v5) [(*17*)](https://www.zotero.org/google-docs/?kHYaOi) was the program of choice as it is a highly accurate and sensitive structural alignment program. To search against the entirety of the AlphaFold database (~200 million structures) [(*14*)](https://www.zotero.org/google-docs/?tWNzXm), DALI’s slow performance presented a major bottleneck. To circumvent this, the search was performed against the ~2.3 million cluster representatives generated by clustering the AlphaFold database with Foldseek [(*20*)](https://www.zotero.org/google-docs/?79EzTM).

To further expedite the structural-search process, the ~2.3 million cluster representatives were subdivided into batches that each contained 1000 structures, resulting in a total of 2304 batches. As DALI requires a four character alphanumeric code for input file names, the structural files downloaded from the AlphaFold database were renamed according to the following scheme. Taking advantage of the case-sensitivity of DALI, files were renamed using a base-62 numbering system (composed of 0–9, A–Z, a–z) where the first two characters indexed the batch number, and the last two characters indexed a structure within the batch. Following the generation of the DALI-search ready database, the curated HEPN dimer cores of known Cas13 proteins were used as queries for DALI comparisons against every batch of target structures. This process was parallelized across all batches, with each job typically using multiple threads. Searches against a single batch usually took less than a minute, and the full search typically finished in less than ten minutes on the UCSF-Wynton high-performance compute cluster when using ten or more threads.

After completing DALI-searches against the AlphaFold database, only hits with the signature Rx4H motif of HEPN domains and a DALI Z-score greater than 6 were retained. Finally, only hits that also bear an intramolecular HEPN dimer were considered as Cas13 candidates. Sequences of Cas13 candidates were retrieved and used as input for PSI-BLAST [(*45*)](https://www.zotero.org/google-docs/?TvD5EJ) and HMMsearch [(*46*)](https://www.zotero.org/google-docs/?LUsac6) against the NCBI NR database [(*47*)](https://www.zotero.org/google-docs/?osHwgk) using default settings. Following this sequence-based enrichment step, contigs encoding the Cas13 candidates were downloaded, and annotated for CRISPR arrays using the CRT tool (v1.2) [(*48*)](https://www.zotero.org/google-docs/?MoFTmJ). This analysis led to the identification of the Cas13an systems characterized in this study.

Structural and phylogenetic analysis of Cas13 proteins and HEPN proteins

For phylogenetic analysis of Cas13 proteins, sequences were obtained from the referenced study [(*13*)](https://www.zotero.org/google-docs/?qDnl4S) and their structures were predicted using ColabFold with three recycles, omitting amber relaxation [(*43*)](https://www.zotero.org/google-docs/?Dyzvy9). The ColabFold structures were filtered for models with an average pLDDT greater than 70, and the resulting dataset was used for all-to-all comparisons using DALI to generate pairwise structural alignments. DALI outputs were fed into a custom Python script that converted DALI alignments into pairwise sequence alignments and merged them to create multiple sequence alignments via T-COFFEE (v13.46.1.b8b01e06) [(*49*)](https://www.zotero.org/google-docs/?ky8Q7c). To ensure maximal accuracy, a DALI-generated structural dendrogram was used as the guide tree during the merging process. This structurally informed multiple sequence alignment was used as input for phylogenetic analysis with IQTREE (v1.6.6) using automatic model selection and 1000 bootstrap replicates [(*50, 51*)](https://www.zotero.org/google-docs/?hymh4C). The resulting tree of Cas13 proteins was rooted using a non-Cas13 protein that also forms an intramolecular HEPN dimer and has HEPN domains that are rearranged like Cas13s. Cas13 protein alignments and phylogenetic tree are found in supplementary data S1.

For the analysis of HEPN domains within Cas13an proteins, representative models of Cas13an proteins identified in this study were generated using ColabFold with three recycles without amber relaxation. After filtering for models with an average pLDDT greater than 70, individual HEPN1 and HEPN2 domains were extracted in ChimeraX. Each domain was then used as a query for the DALI structural-search pipeline. Only hits above a DALI Z-score above 6 that appeared at least twice in searches of either HEPN1 or HEPN2 domains were retained for further analysis. Hits that were either not in the range of 150–450 amino acids or lacked the Rx(4,5)H motif in the expected helix region were also discarded. A final manual examination of the structures was conducted to ensure the inclusion of only true HEPN proteins. Alignments and phylogenetic trees were subsequently generated as done above for Cas13 phylogenetic analysis. The final tree was rooted using a HEPN protein that lacks rearrangements in the HEPN domain conserved among Cas13 HEPN domains and their closely related homologs. The HEPN protein alignments and phylogenetic tree are found in supplementary data S1.

Molecular cloning and plasmid construction

gBlocks of minimal CRISPR-Cas13an loci that included a Cas13an, a mini CRISPR array containing one repeat-spacer-repeat, and the intergenic regions linking them, were ordered from Twist Biosciences and cloned into arabinose-inducible expression vectors (pBAD) via Golden Gate cloning. Vectors of this design were utilized in all *E. coli* cell-based assays, including small RNA-sequencing, green fluorescent protein (GFP) depletion, phage restriction, mismatch, and flanking sequence preference screening assays. For Cas13an protein expression, plasmids were modified to include a fusion of a 10xHIS-MBP-TEV tag at the N-terminus. No other changes were made to the vector configuration for protein expression. Sequences of constructs used in this study, as well as DNA oligos used in their assembly, are found in supplementary data S2.

Small RNA-sequencing and analysis

For heterologous RNA expression, *E. coli* was transformed with plasmids encoding minimal Cas13an loci and grown on LB agar plates containing 50 µg/mL ampicillin overnight. Multiple colonies were picked and inoculated in 5mL liquid LB culture containing 50µg/mL ampicillin. Cultures were incubated at 37ºC and 200 RPM until reaching an OD600 of 0.6. At this point, gene expression was induced by the addition of arabinose to a final concentration of 10 mM and the temperature was reduced to 16ºC. Following overnight growth, total RNA from cells was extracted using a hot formamide method [(*52*)](https://www.zotero.org/google-docs/?Ci6W5v), in which pelleted *E. coli* were resuspended in an 18 mM EDTA and 95% formamide solution and lysed by incubating at 65ºC for 5 minutes. After centrifugation to pellet cell debris, the supernatant containing total RNA was purified using the RNA Clean & Concentrator-5 Kit (Zymo Research R1013) following manufacturer protocols. Approximately 200 ng of purified total RNA was subjected to rRNA depletion using the NEBNext rRNA Depletion Kit for Bacteria (New England Biolabs E7850L) following manufacturer protocols. rRNA-depleted samples were again purified using the RNA Clean & Concentrator-5 Kit. Note that for RNA-sequencing of *in vitro* cleaved RNA products, the rRNA depletion steps were skipped. Instead, RNA products were directly purified from the reaction mixture using the RNA Clean & Concentrator-5 Kit and then treated the same way as rRNA-removed RNA in subsequent processing steps as described below.
 About 20 ng of rRNA-removed RNA samples were used for end repair with QuickCIP (New England Biolabs M0525S) and T4 PNK (New England Biolabs M0201S) treatment using the following procedure. Initially, RNA samples were incubated in a 20 µL reaction containing T4 PNK buffer (New England Biolabs B0201S) and 1 µL QuickCIP at 37ºC for 30 minutes. Following heat-inactivation of QuickCIP (85ºC for 10 minutes), 1 µL T4 PNK was spiked directly into the reaction and further incubated at 37ºC for 30 minutes. Subsequently, ATP was added to a final concentration of 5 mM, and the reaction mixture was incubated at 37ºC for an additional 30 minutes. Following end repair, RNA samples were again purified using the RNA Clean & Concentrator-5 Kit . The purified RNA was then subjected to library preparation using the Collibri™ Stranded RNA Library Prep Kit for Illumina™ Systems (Invitrogen A38994024) following manufacturer protocols for small RNA-sequencing. The amplified cDNA was size-selected and purified via gel extraction from E-Gel™ EX 4% Agarose Gels (Invitrogen G401004) and quantified using the KAPA Library Quantification Kit (Roche KK4873). Sequencing was performed by the Innovative Genomics Institute Next-Generation Sequencing Core using an Illumina NextSeq 1000/2000 P2 v3 kit (Illumina), configured for 2×150 bp paired-end reads. *In vivo* sequencing assays were performed once, and *in vitro* processing sequencing assays were performed with two technical replicates. Raw reads from the *in vivo* assays were trimmed and merged using BBmerge (v38.84) [(](https://www.zotero.org/google-docs/?avtJB3)*53*), while fastp (v0.23.4) [(*54*)](https://www.zotero.org/google-docs/?i2hkJF) was used for reads from *in vitro* assays. Finally, the processed reads were aligned to the reference loci using Geneious mapper with low sensitivity. For *in vivo* small RNA-sequencing assays shown in supplementary figures, an additional filtering step was performed. Following read mapping to the reference plasmid, reads specifically mapping to the CRISPR array region were extracted, size filtered for a length of at least 40, and realigned to the CRISPR array using Geneious mapper with low sensitivity.

GFP depletion assays

GFP depletion assays were conducted with at least three biological replicates. In experimental conditions, the spacer was reprogrammed to target GFP, whereas in control experiments, the spacer was a Golden Gate cloning site of the same length. For the assay, 100 ng of the Cas13an plasmid was co-transformed with either 100 ng of GFP or red fluorescent protein (RFP) expressing plasmids into *E. coli* NEB-10B strains (New England Biolabs C3020K). Following a one hour recovery, the transformed cells were serially diluted, and 5 µL of the dilutions were spot plated on LB agar containing 100 µg/mL ampicillin, 50 µg/mL kanamycin, and optionally 10 mM arabinose for induction of Cas13an. Plates were incubated overnight at 37ºC, and fluorescence intensity was measured to assess depletion of the fluorescent proteins.

Phage propagation and scaling

Phages were propagated using standard protocols either in LB media or LB top agar overlays (0.7%), LB top agar overlays (0.35%) (Goslar, G17), or LB top agar overlays (0.7%) supplemented with 1mM CaCl2 (M13, MS2). Phages EdH4, MM02, N4, PTXU04, SUSP1, T4, T5, and T7 were propagated on *E. coli* BW25113. Other phages were propagated on *E.coli* MC1000 (Goslar), *E.coli* ECOR47 (G17) or *E.coli* DH5α F’Iq (New England Biolabs C2992) with 1 mM CaCl2 (MS2, M13). All phages were titrated by spotting 2 µL of 10-fold serially diluted phage in SM buffer (Teknova S0249) on *E. coli* BW25113 in a top agar overlay (with modifications denoted above). Phages SUSP1 and Goslar were gifts from Drs. Sankar Adhya and Joseph Pogliano, respectively. Phages EdH4, G17, MM02, and PTXU04 were purchased from the DSMZ culture collection. Phages M13 and MS2 were purchased from the ATCC.

Efficiency of plaquing assays

Bacteriophage assays were conducted using a modified double agar overlay method with modifications noted in “phage propagation and scaling” above. All assays were conducted with at least three biological replicates. Each assay involved *E. coli* harboring a specific CRISPR-Cas13an locus with a reprogrammed spacer, grown overnight at 37°C and 250 RPM. To perform plaque assays, 100 µL of saturated culture was mixed with molten LB top agar containing inducers and antibiotics (to a final concentration of 10 mM arabinose and 100 µg/mL carbenicillin). Overlays were dried for 15 minutes before 2 µl spots of 10-fold serial dilutions of phages in SM buffer were applied and dried for 10 minutes. The plates were incubated overnight at 30°C for 16 hours. After incubation, plaques were scanned, and plaque-forming units (p.f.u.s) were counted. In cases with lysis but no countable plaques, the highest dilution showing lysis was estimated as 1 p.f.u. The efficiency of plaquing (EOP) was calculated by comparing the average p.f.u. of each condition to that of a non-targeting control.

Protein production and purification

Cas13an expression vectors were transformed into One Shot™ BL21-AI™ Chemically Competent *E. coli* (Invitrogen C607003) and grown overnight at 37°C on LB agar plates containing 100 µg/mL ampicillin. Using the same ampicillin concentration, 50 mL LB cultures were inoculated with single colonies picked from the LB agar transformation plates. Cultures were grown at 37°C and 240 RPM overnight at. Subsequently, 1 L of 2XYT media containing 100 µg/mL ampicillin was inoculated with 25 mL of the overnight starter culture and incubated at 37°C and 150 RPM. Once reaching an OD600 of 0.6, cultures were chilled on ice for 30 minutes before the addition of 10 mM arabinose, and incubated overnight at 16°C and 120 RPM. After collecting cells by centrifugation, pellets were resuspended in wash buffer (300 mM NaCl, 1 mM TCEP, 50 mM imidazole, 100 mM Tris pH 8) and lysed via sonication. The lysate was clarified by centrifugation, and the soluble fraction was purified using a 5 mL Ni-NTA Superflow Cartridge (Qiagen 30761) on an ӒKTA pure™ chromatography system (Cytiva) using elution buffer (300 mM NaCl, 1 mM TCEP, 500 mM imidazole, 100 mM Tris pH 8). The eluted product from Ni-NTA purification was further purified using a 5mL HiTrap Heparin column (Cytiva 17040703) on an ӒKTA. The purified proteins were concentrated and subjected to size exclusion chromatography (SEC) using a Superdex® 200 Increase 10/300 GL (GE Healthcare GE28-9909-44) with wash buffer. Protein concentrations were measured with a NanoDrop 8000 Spectrophotometer (Thermo Scientific) and concentrated up to 10 mg/mL using Amicon® Ultra Centrifugal Filters (Millipore Sigma UFC905024). Protease cleavage steps to remove the expression tags were not performed as MBP fusion substantially improved solubility. Concentrated proteins were kept at 4°C, snap frozen in liquid nitrogen, and stored at -80°C. Binary or ribonucleoprotein complex (RNP) was prepared by incubating refolded crRNA (generated by heating to 67°C for 10 minutes and gradually cooling to room temperature) and Cas13an protein at a 1:1 ratio in RNP assembly buffer (50 mM HEPES pH 6.8, 150 mM KCl, 5 mM MgCl_2_, 10% glycerol, 1 mM TCEP) for 10 minutes at 37°C. For the EDTA condition for the target cleavage assay, 5 mM MgCl_2_ was replaced with 10 mM EDTA in the RNP assembly buffer, and the final total MgCl_2_ concentration in solution was less than 20 nM.

*In vitro* generation and labeling of RNA substrates

All RNAs used in biochemical assays were transcribed *in vitro*. DNA templates for *in vitro* transcription were generated via either oligo-extension amplification or by amplifying plasmids encoding CRISPR-Cas13an systems with KAPA HiFi polymerase (Roche KK2602) or PrimeStar GXL polymerase (Takara R050A). *In vitro* transcription was initiated by adding either DNA purified using the Monarch® PCR & DNA Cleanup Kit (5 μg) (New England Biolabs T1030S) or the PCR reaction directly into the transcription reaction buffer. The transcription reaction buffer contained 30 mM Tris, 25 mM MgCl_2_, 10 mM DTT, 0.001% Triton, 2 mM Spermidine, 5 mM rNTP, and ~1 uM T7 polymerase. Transcription reactions were incubated at 37°C for 3-4 hours, before quenching by 2X RNA loading buffer (New England Biolabs B0363S). Transcribed RNAs were size separated and extracted via 8% denaturing urea-polyacrylamide gel electrophoresis (PAGE) followed by overnight soaking in DEPC water at 4 °C. Following concentration and multiple DEPC water washing steps using Amicon® Ultra Centrifugal Filters, the purified RNA was stored -80°C. In some instances, HiScribe® T7 High Yield RNA Synthesis Kit (New England Biolabs E2040S) was used to generate RNAs. These samples were directly purified using Monarch® RNA Cleanup Columns (500 µg) (New England Biolabs T2057L) following manufacturer protocols.

To label RNA substrates, approximately 1 nmol of RNA was processed in a 20 µL reaction as follows. In T4 PNK buffer, RNA was treated with 1 µL of QuickCIP at 37°C for 30 minutes. After heat inactivation at 85°C for 10 minutes, 2 µL of T4 PNK and 1 µL of gamma-thiol ATP (10 µg/µL) (Sigma Aldrich A1388) was added, followed by another 30 minute incubation step at 37°C. Then, 5 µL of a 10 µg/µL thiol-reactive dye (Biotium 91028) was added, and the mixture was incubated at 65°C for 30 minutes. Following the labeling step, RNA samples were purified using the RNA Clean & Concentrator-5 Kit following manufacturer protocols.

*In vitro* cleavage assays

For *cis*-cleavage reactions, 500 nM RNP was combined with 250 nM 5’-fluorescein (FAM)-labeled substrate in cleavage buffer (50 mM HEPES pH 6.8, 50 mM KCl, 5 mM MgCl_2_, 10% glycerol, 1 mM TCEP) and incubated at 37°C. Reactions were quenched by the addition of 2X RNA loading buffer (New England Biolabs B0363S) and heating to 95°C for 1 minute. Samples were separated on 15% denaturing urea-PAGE gels. Gels were imaged on an Amersham Typhoon scanner (GE Healthcare) using a 488 nm laser, and bands were quantified in Bio-Rab ImageLab 6.1. The *cis*-cleavage curves from four technical replicates were fitted to a one-phase-decay model in Prism 10 (GraphPad) to derive the rate of cleavage.

For *trans*-cleavage reactions, 500nM RNP was combined with 250nM of unlabeled target RNA or non-target RNA, and 250nM of RNaseAlert fluorophore-quencher substrate (Integrated DNA Technologies 11-04-03-03) in cleavage buffer. The reaction was initiated on ice, and immediately moved to a BioTek Cytation 5 plate reader (Agilent) set at 37°C, and allowed to proceed for an hour. Fluorescence intensity was monitored using 480 nm excitation and 520 nm emission. *Trans*-cleavage assays were performed with two technical replicates.

Mismatch Tolerance Assay and Flanking Sequence Preference Assay

All library-based *E. coli* assays described in this section were performed with two biological replicates. For mismatch tolerance assays, plasmid libraries containing CRISPR-Cas13an2 loci with mismatched spacer sequences targeting the *kanR* gene were co-transformed with *kanR* expressing plasmids into *E. coli* NEB-10B strains. For flanking sequence preference assays (also known as protospacer flanking sequence or PFS assays), a plasmid containing CRISPR-Cas13an2 loci targeting the 5’ untranslated region of the *kanR* gene was transformed into *E. coli* NEB-10B strains already containing the flanking sequence library plasmid. Post-transformation, cells were recovered for four hours and then plated on LB agar with 100 µg/mL ampicillin, either 200 µg/mL (in mismatch assay) or 400 µg/mL (in flanking sequence preference assay) kanamycin, and optionally 10 mM arabinose to induce Cas13an expression. After incubating at 37°C for 16 hours, plasmid DNA was extracted from surviving cells and prepared for sequencing by PCR amplification using Illumina adaptor primers. Sequencing was performed by the Innovative Genomics Institute Next-Generation Sequencing Core using an Illumina NextSeq 1000/2000 P2 v3 kit (Illumina), configured for 2×150 bp paired-end reads with an average depth of approximately few million reads per sample. Raw reads were trimmed and merged using fastp (v0.23.4), then aligned to reference loci using BWA-MEM (v0.7.17) [(*54*)](https://www.zotero.org/google-docs/?N8rxyP). Quality amplicons were isolated with samtools (v1.6) [(*55*)](https://www.zotero.org/google-docs/?YDR5dh) bam2fq.

Mismatch tolerance was quantified using a custom Python script and regular expressions to extract the library of 945 spacers. Spacer frequencies were normalized to alignment coverage to account for variations in sequencing depth and library preparation. Log2-fold changes were then calculated relative to each plasmid library. Inactive guides were identified as those with log2-fold changes exceeding the 99.9999% confidence interval for the minimum log2-fold change in the uninduced samples. The percentage of inactive guides with mismatches at each position was quantified and visualized to assess the positional effects of mismatches on target recognition.

Flanking sequence preference was characterized similarly, after introducing 3 nucleotide (nt) randomized regions to the 5’ and 3’ flanks of the target sequence in the *kanR* plasmid. Flanking sequence frequencies were tabulated from FASTQs and normalized for sequencing depth. The log2-fold change of each sequence in the flanking sequence library was calculated relative to its average frequency observed in competent cells. The 5’ and 3’ flanking sequence preferences were assessed separately by establishing a 99.9999% confidence interval for the minimum log2-fold change in non-targeting samples. No statistically significant flanking sequence preference depletion was observed compared to non-targeting negative controls.

*In vitro* processing assays

All processing assays were performed with at least three technical replicates. RNA substrates were incubated at 67°C for 10 minutes, and gradually cooled to room temperature to ensure proper folding. Processing of the pre-crRNA substrate (134nt) was initiated by mixing 500 nM Cas13an with 500 nM pre-crRNA substrate in cleavage buffer. Following incubation at 37°C for 6 hours (with the exception of sequenced pre-crRNA products, which were incubated overnight), reactions were quenched by addition of 2X RNA loading dye and heating to 95°C for 1 minute. Quenched samples were size separated on 15% denaturing urea-PAGE gels and stained with SYBR^TM^-Gold (ThermoFisher Scientific S11494). Processing assays of full-length crRNA containing a 30nt spacer was performed under identical conditions using a 5’-FAM-labeled RNA substrate. The alkaline hydrolysis ladders were created by incubating 5’-FAM-labeled RNA in alkaline hydrolysis buffer (50 mM sodium carbonate, pH 9.4, 1 mM EDTA) at 95°C for 5 minutes, quenched by 2X RNA loading buffer and stored at -80°C. Gels were scanned on an Amersham Typhoon scanner using a 488 nm laser to visualize both FAM and SYBR-GOLD staining.

**Figure S1**


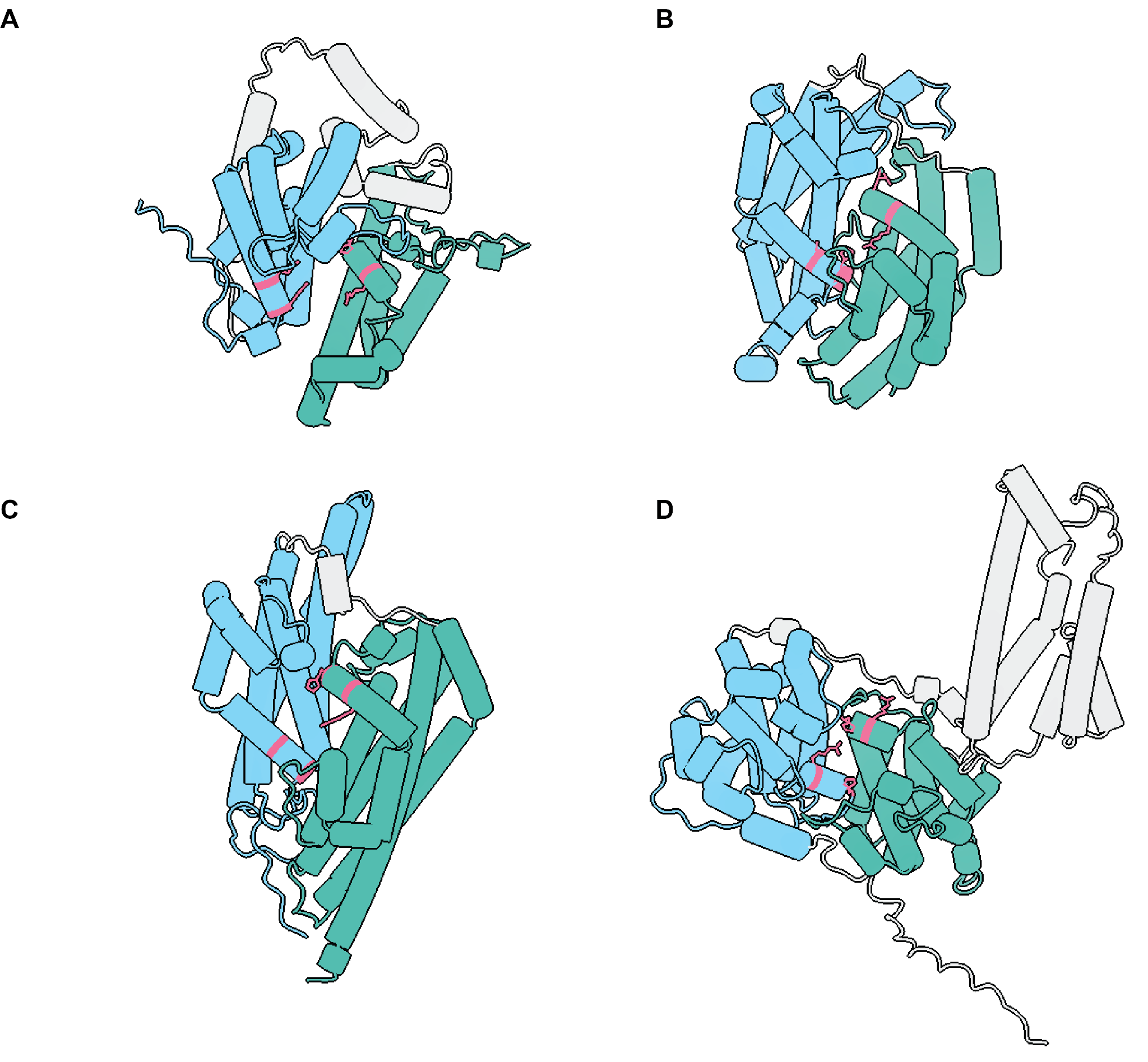


**Fig. S1. Representative proteins with intramolecular HEPN dimers identified through structural homology in this study**
Blue represents N-terminal HEPN domain (HEPN1), green represents C-terminal HEPN domain (HEPN2), gray represents insertions linking the two HEPN domains, and red represents the Rx(4,5)H catalytic motif. **(A)** Representative Cas13an protein identified from DALI-search against clustered AlphaFold database (AFDBID: A0A7C5SD50). **(B)** “HEPN_Cthe2314 domain-containing protein” (AFDBID: G7MBT4) found in prokaryotes that has canonical organization of HEPN domains. **(C)** Uncharacterized protein (AFDBID: A0A1Y6ANL6) found in prokaryotes that has canonical organization of HEPN domains. **(D)** DUF4209 domain-containing protein (AFDBID: A0A7M7T5H2) found in eukaryotes. This protein has identical HEPN domain rearrangement found in Cas13s. Both variants with and without insertions linking the two HEPN domains were identifiable.

**Figure S2**


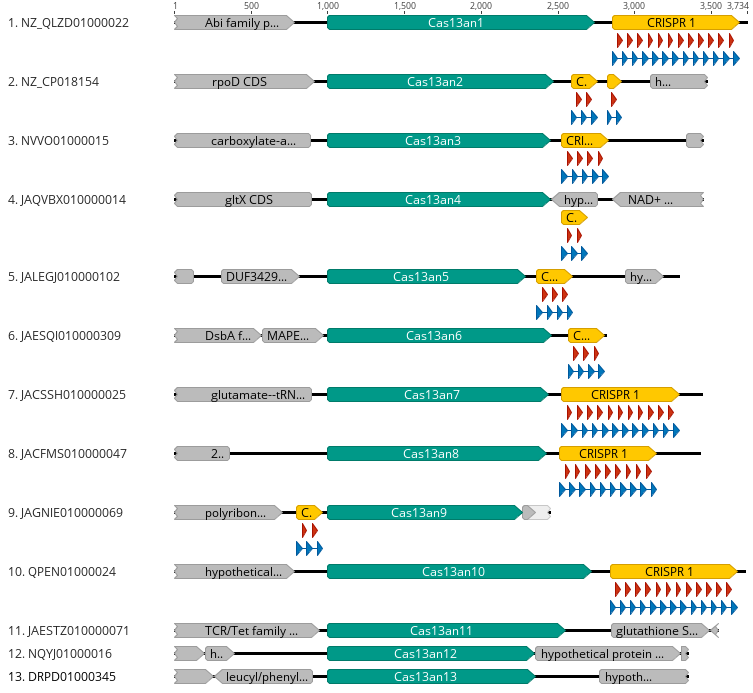


**Fig. S2. Genomic context of CRISPR-Cas13an systems**CRISPR-Cas13an systems encode only the *cas13an* effector gene and the CRISPR array, which is usually found immediately downstream.

**Figure S3
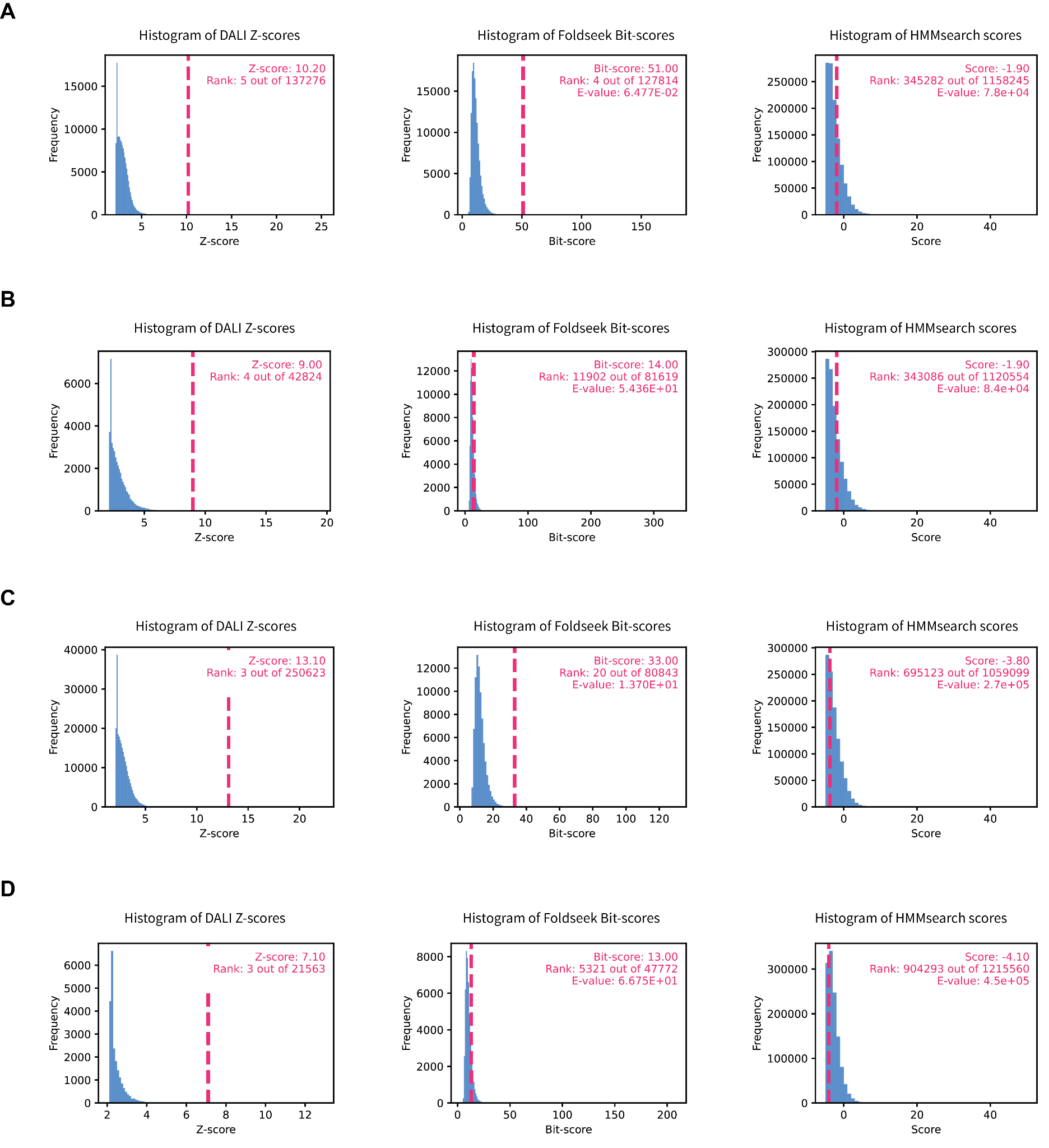
**

**Fig. S3. Benchmarking of DALI, Foldseek, and hidden Markov model searches**Benchmarking of different search methods using different Cas13 as queries: **(A)** Cas13a **(B)** Cas13b **(C)** Cas13c **(D)** Cas13d. Dotted line indicates where the newly discovered Cas13an hit was found among the output lists. Within the histogram, the respective search-score, ranking, and E-values where applicable are displayed.

**Figure S4**

**
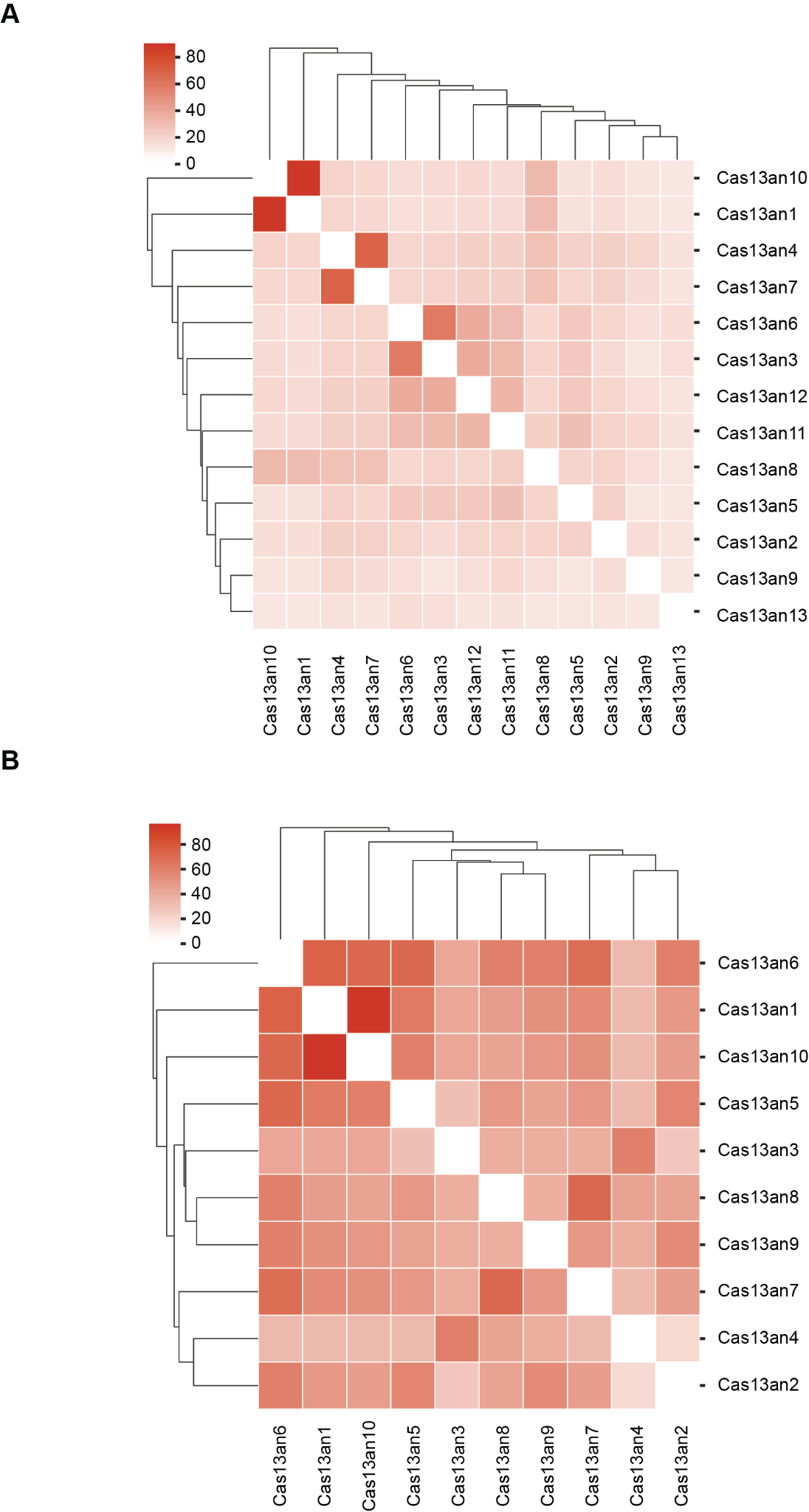
**

**Fig. S4. Heatmap of Cas13an protein and CRISPR-repeat sequence comparisons**.
**(A)** Heatmap showing % sequence identity comparisons across all Cas13an protein sequences identified in this study. **(B)** Heatmap showing % sequence identity comparisons of Cas13an CRISPR-repeat sequences tested in this study.

**Figure S5**

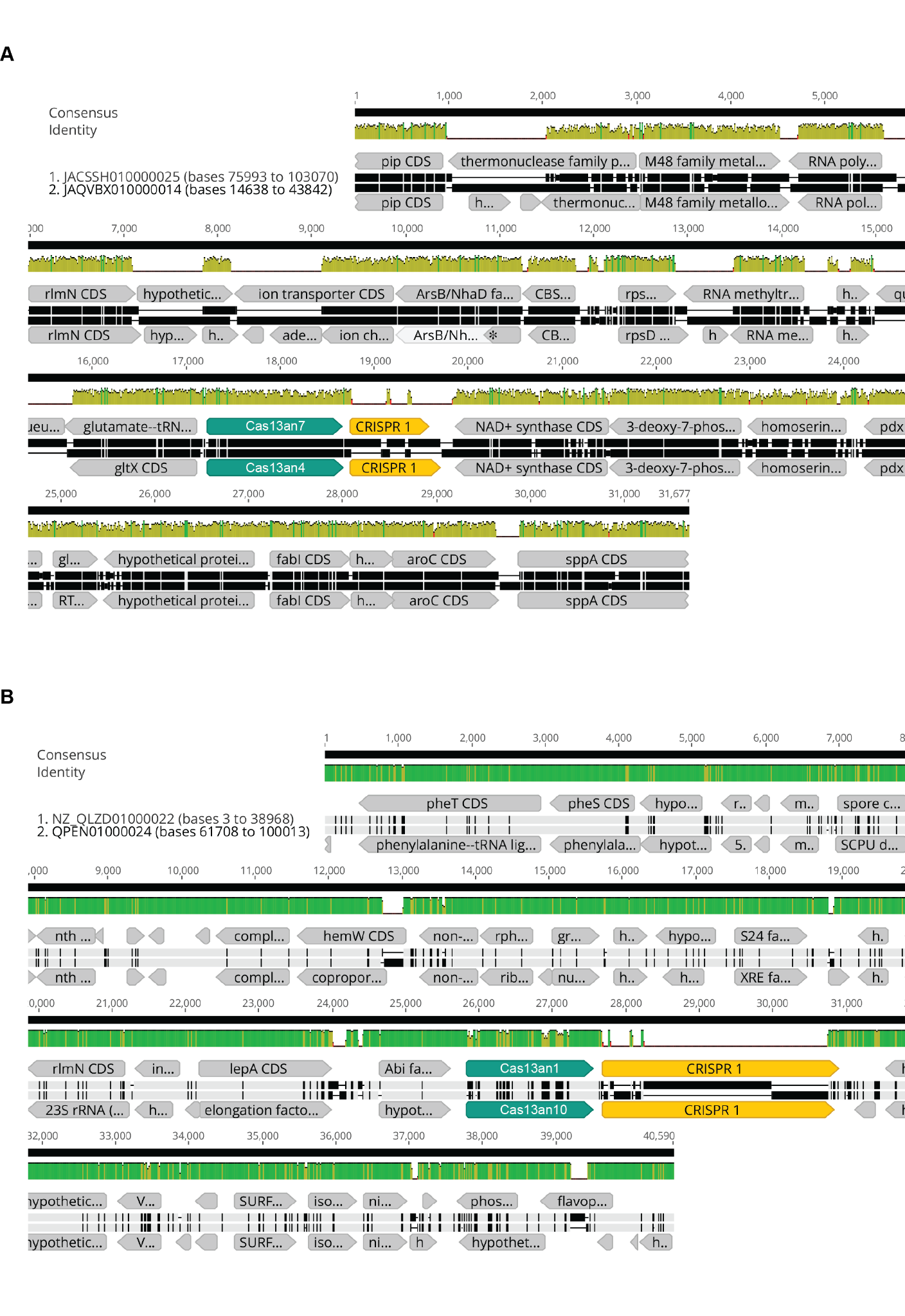


**Fig. S5. CRISPR-Cas13an systems actively acquire spacers in the absence of acquisition machinery in *cis*.**Mauve-whole genome alignment of two different pairs of contigs **(A)** and **(B)** encoding closely related Cas13an orthologs. Despite lacking genes associated with spacer acquisition (*cas1*, *cas2*, *cas4*), Cas13an systems are able to acquire new spacers as evidenced by lack of conservation of spacer sequences, but high conservation of the genome outside of the CRISPR array.

**Figure S6**

**
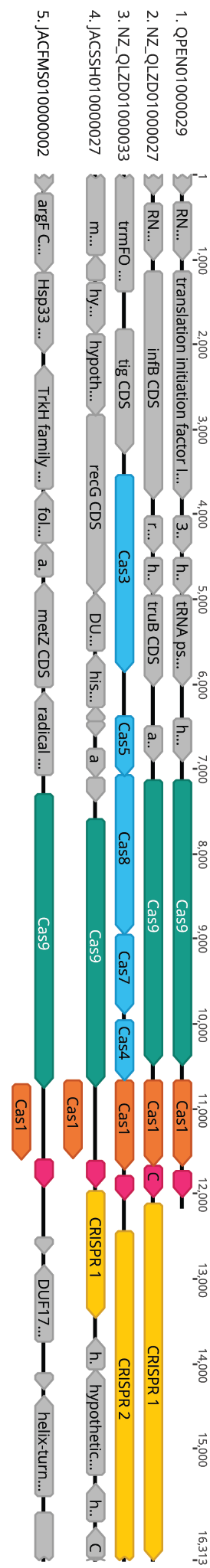
**

**Fig. S6. Putative CRISPR-Cas adaptation modules operating in *trans* of CRISPR-Cas13an systems.**Green indicates *cas9* genes. Blue indicates Type I CRISPR-Cas genes. Orange indicates *cas1* genes. Magenta indicates *cas2* genes. Yellow demarks boundaries of the CRISPR arrays. Note that contig (2) and contig (3) are from the same genome. Protein ID of *cas1* genes on respective contigs: (1) KAA6204368.1, (2) WP_150213413.1, (3) WP_150214154.1, (4) MBE2191857.1, (5) MBW7835230.1.

**Figure S7**

**
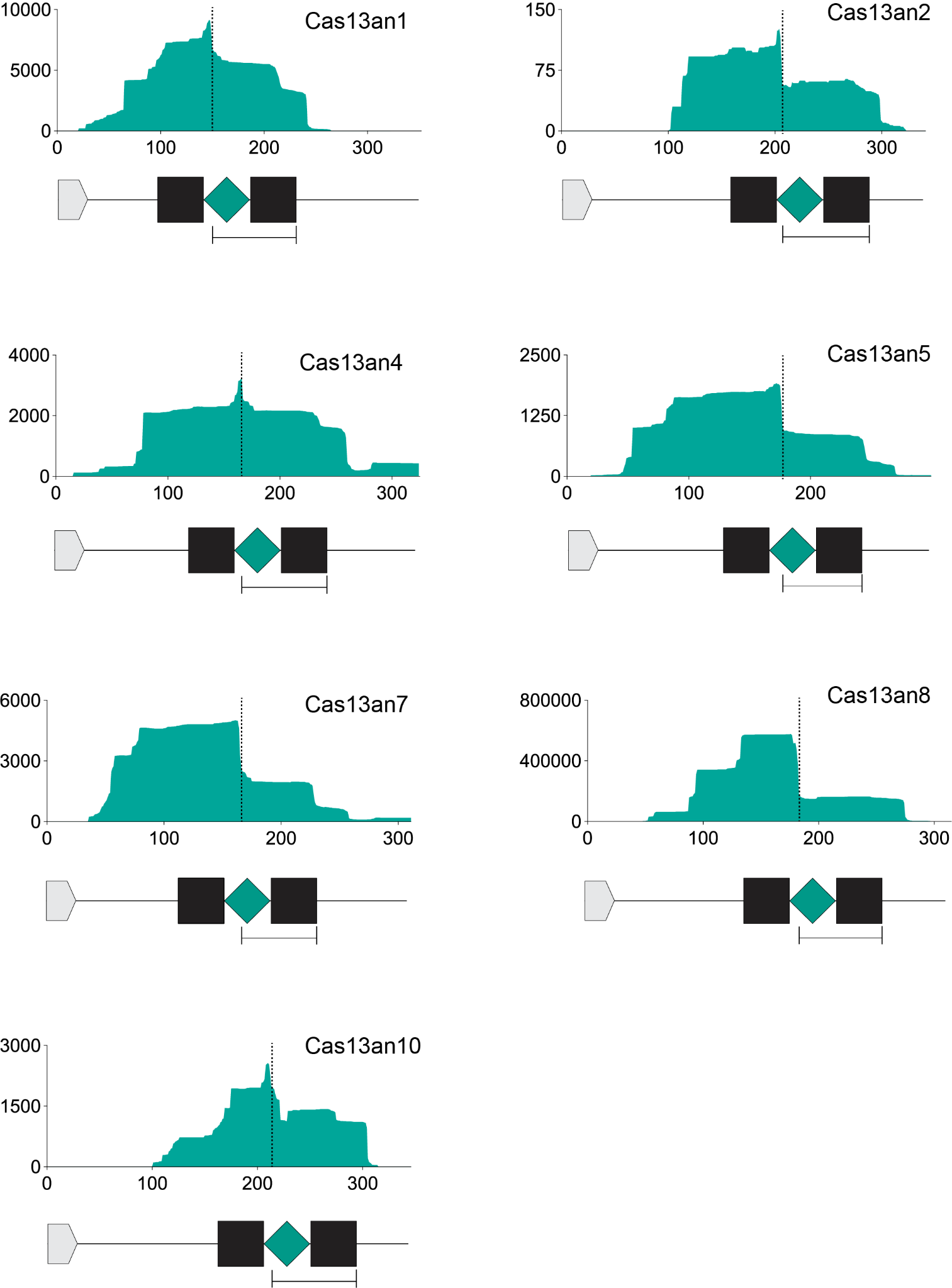
**

**Fig. S7. Heterologous expression of Cas13an associated CRISPR RNAs (crRNAs) in *E coli.***

Coverage map of small RNA-sequencing reads mapped to the CRISPR array. Black square denotes CRISPR-repeat, and green diamond denotes spacer sequence.

**Figure S8**

**
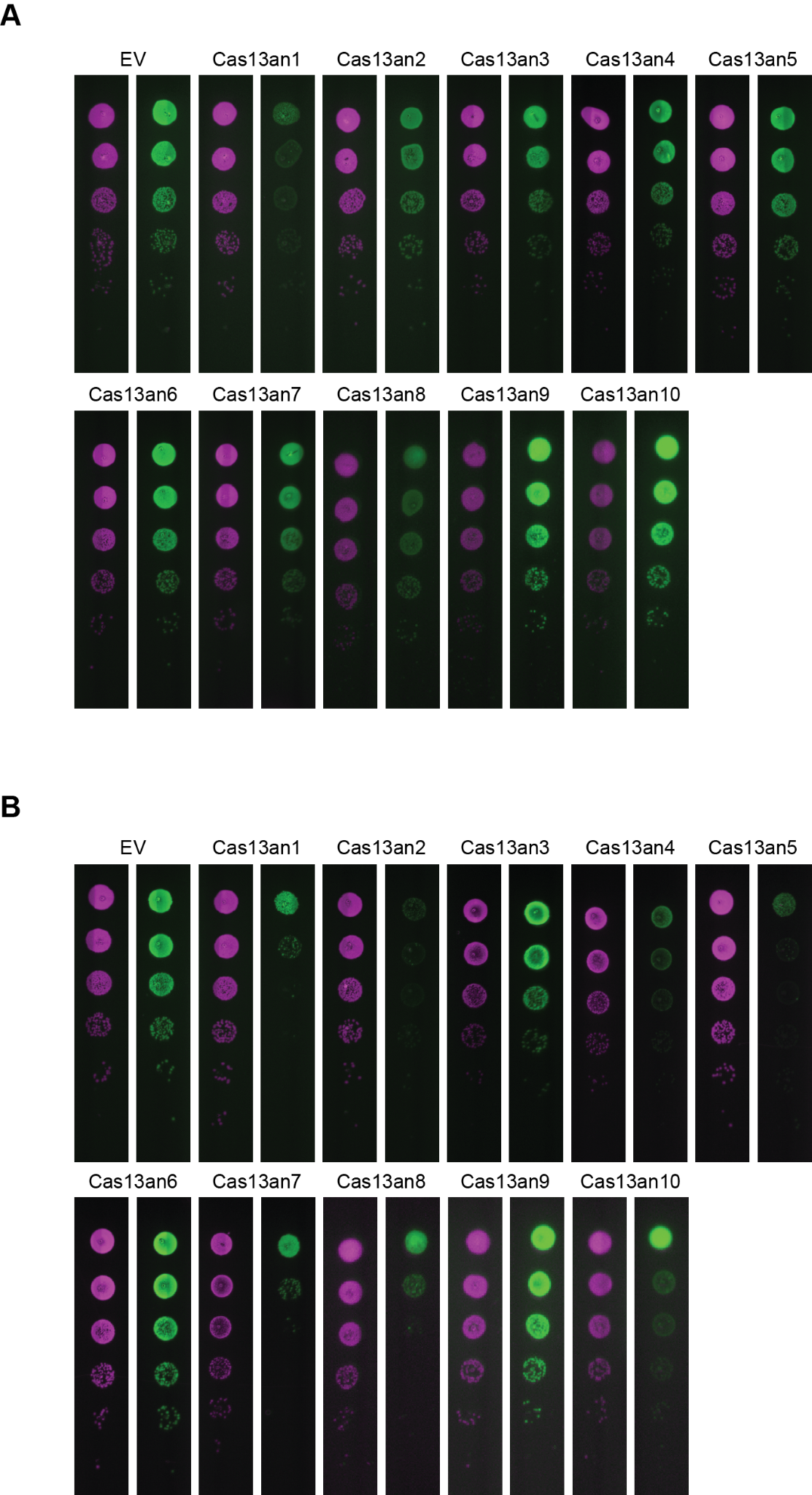
**

**Fig. S8. Scan images of green fluorescent protein (GFP) depletion assays.**

Plasmid assays of all CRISPR-Cas13an systems tested in *E. coli*. EV stands for “Empty Vector” lacking any CRISPR-Cas13an genes. In pairs of images, left shows red fluorescent protein (RFP) and right shows GFP. **(A)** shows non-induced conditions, and **(B)** shows induced conditions. Note that for some Cas13an variants, leaky expression from the pBAD promoter in absence of induction was sufficient for GFP knockdown.

**Figure S9

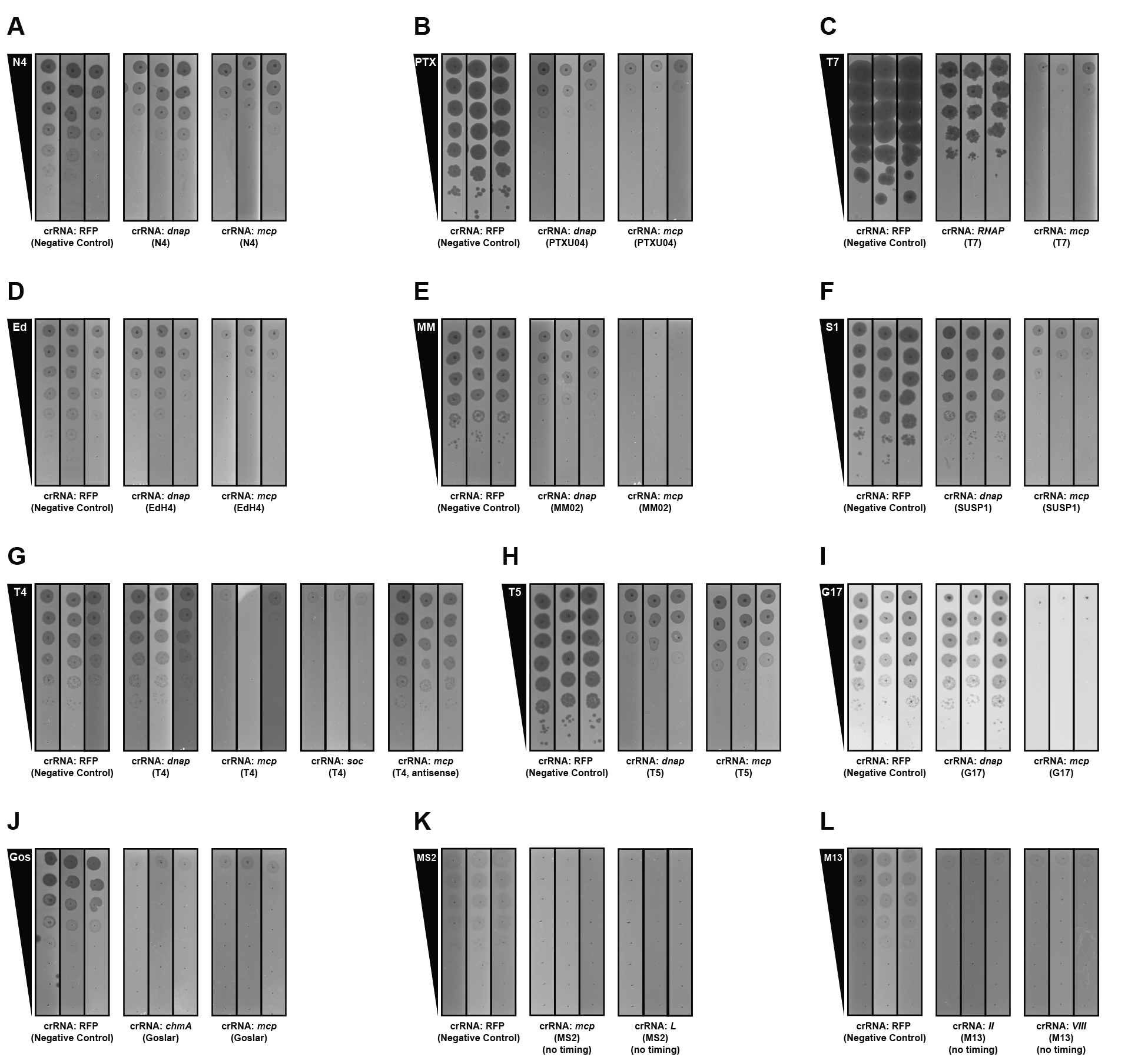
**

**Fig. S9. Complete data for phage plaque assays.**

Plaque assays used to inform phage experiments in Fig. 2D and 2E for **(A)** N4, **(B)** PTXU04, **(C)** T7, **(D)** EdH4, **(E)** MM02, **(F)** SUSP1, **(G)** T4, **(H)** T5, **(I)** G17, **(J)** Goslar, **(K)** MS2, and **(L)** M13. All plaque assays comprise 3 independent biological replicates. Phages are presented in the order as shown in Figure 2E. Phages are representative of individual viral genera, four morphologies of dsDNA phages (*Podovirus* (A-C), *Myovirus* (D-G), *Siphovirus* (H), Jumbo *Myovirus* (I-J)), ssRNA(+) phage (K) and ssDNA(+) phage (L).

**Figure S10

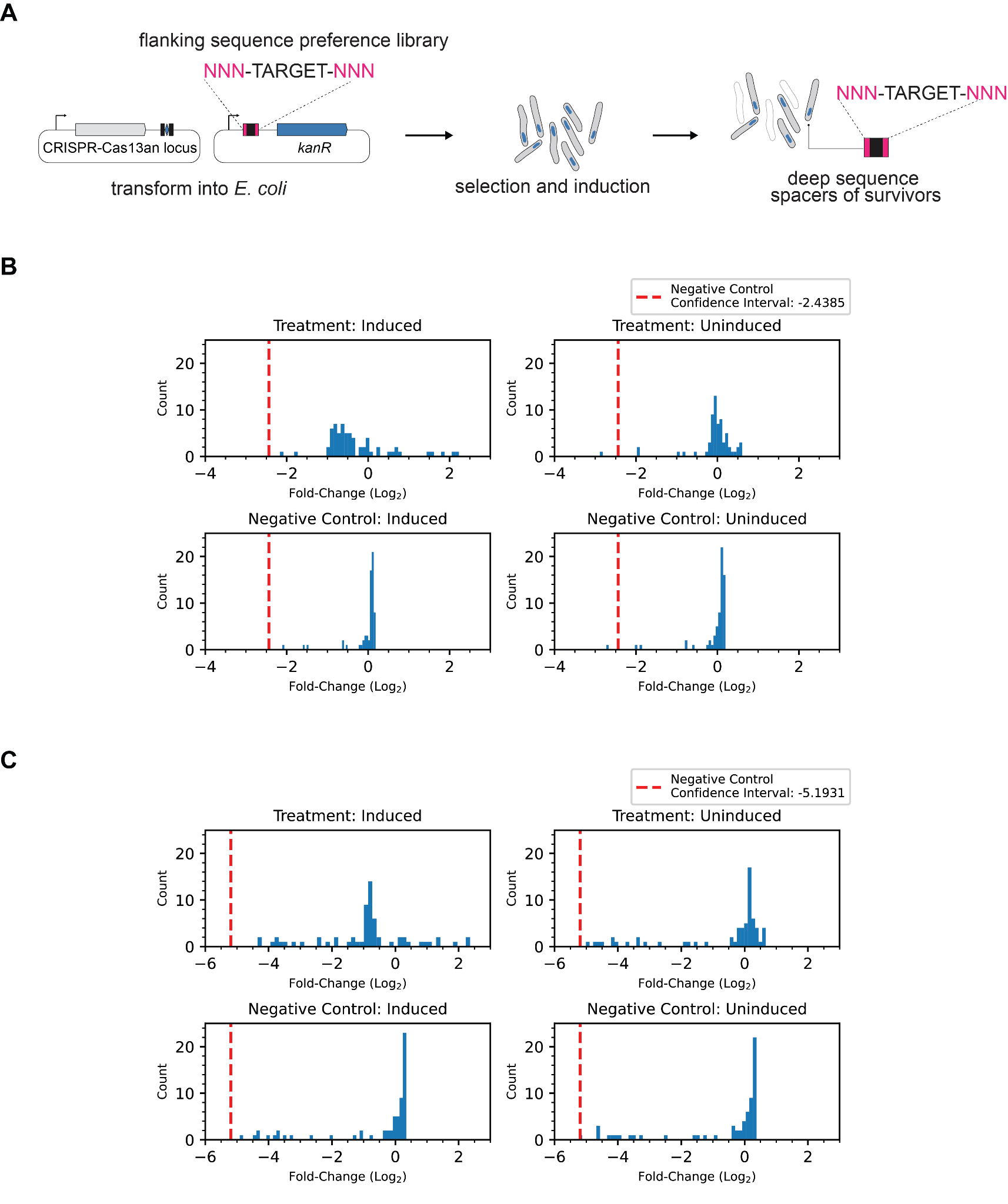
**

**Fig. S10. Results of flanking sequence preference (also known as protospacer flanking sequence or PFS) screening assay.
(A)** Schematic of flanking sequence preference screening assay. A library of target flanking sequences was generated by randomizing 3 nucleotides (nt) on each flank of the target sequence in the *kanR* gene. Following selection on a selective antibiotic plate, preferential depletion of specific motifs were assayed via deep sequencing. **(B, C)** Comparison of log2 fold-change depletion for targeting and non-targeting (negative control) Cas13an2 experiments in induced and uninduced cells. The average log2 fold-change depletion for each condition and the 99.9999% confidence interval for negative-control depletion are displayed for 5’ (B) and 3’ (C) target-flanking sequences. Lack of statistically significant depletion for induced, targeting samples suggests no flanking sequence preference for Cas13an2.

**Figure S11

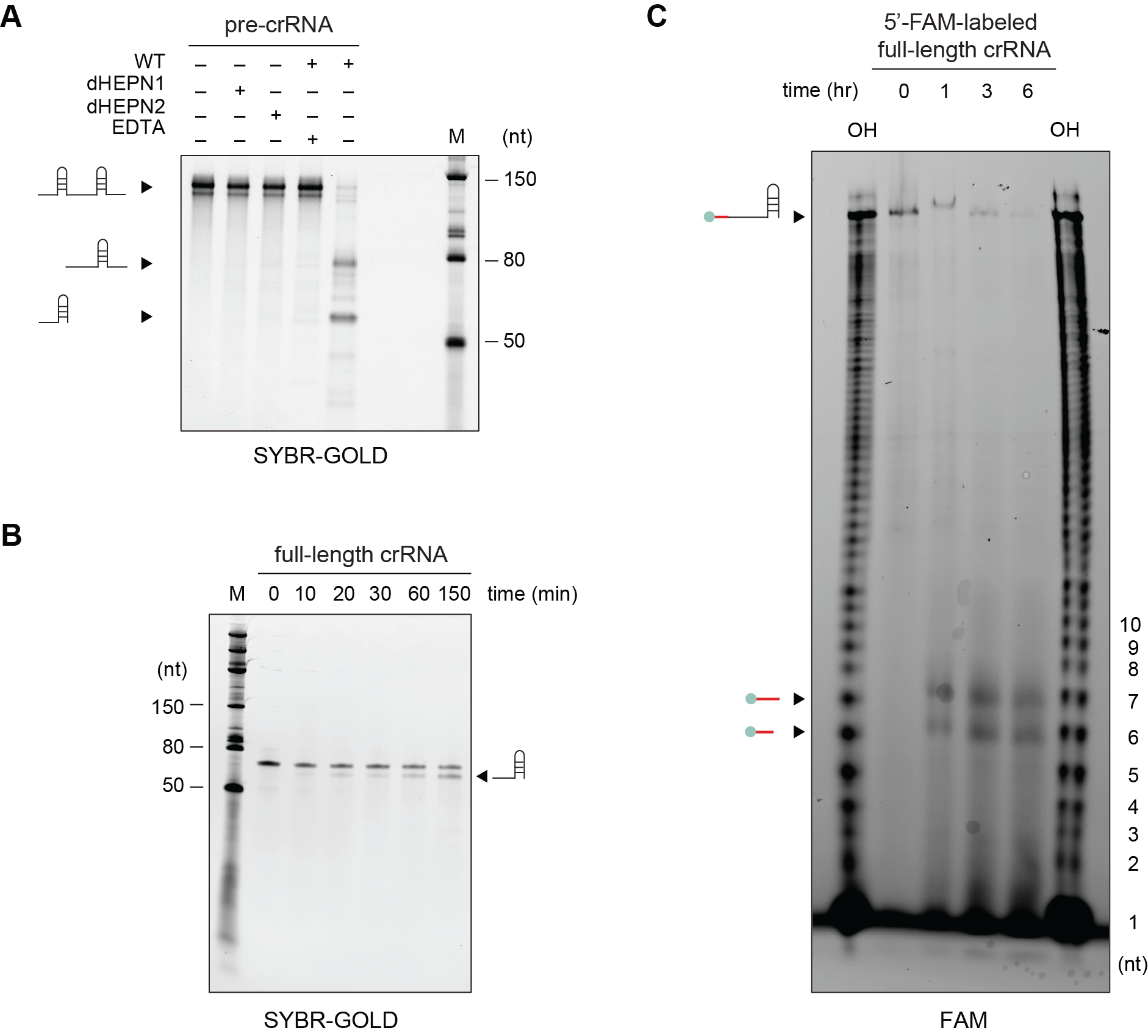
**

**Fig. S11. crRNA processing by Cas13an2.**15% denaturing urea-polyacrylamide gel electrophoresis (PAGE) gels depicting Cas13an processing of pre-crRNA and full-length crRNA. **(A)** Larger crop image of Fig. 4B that shows processing of pre-crRNA. M, ssRNA ladder. Visualized by SYBR-GOLD staining. **(B)** Larger crop image of Fig. 4C right panel that demonstrates processing of full-length crRNA. M, ssRNA ladder. Visualized by SYBR-GOLD staining. **(C)** *In vitro* RNA processing of 5’-fluorescein (FAM)-labeled full-length crRNA. OH, alkaline hydrolysis ladder. Visualized by imaging FAM labels.

**Figure S12**

**
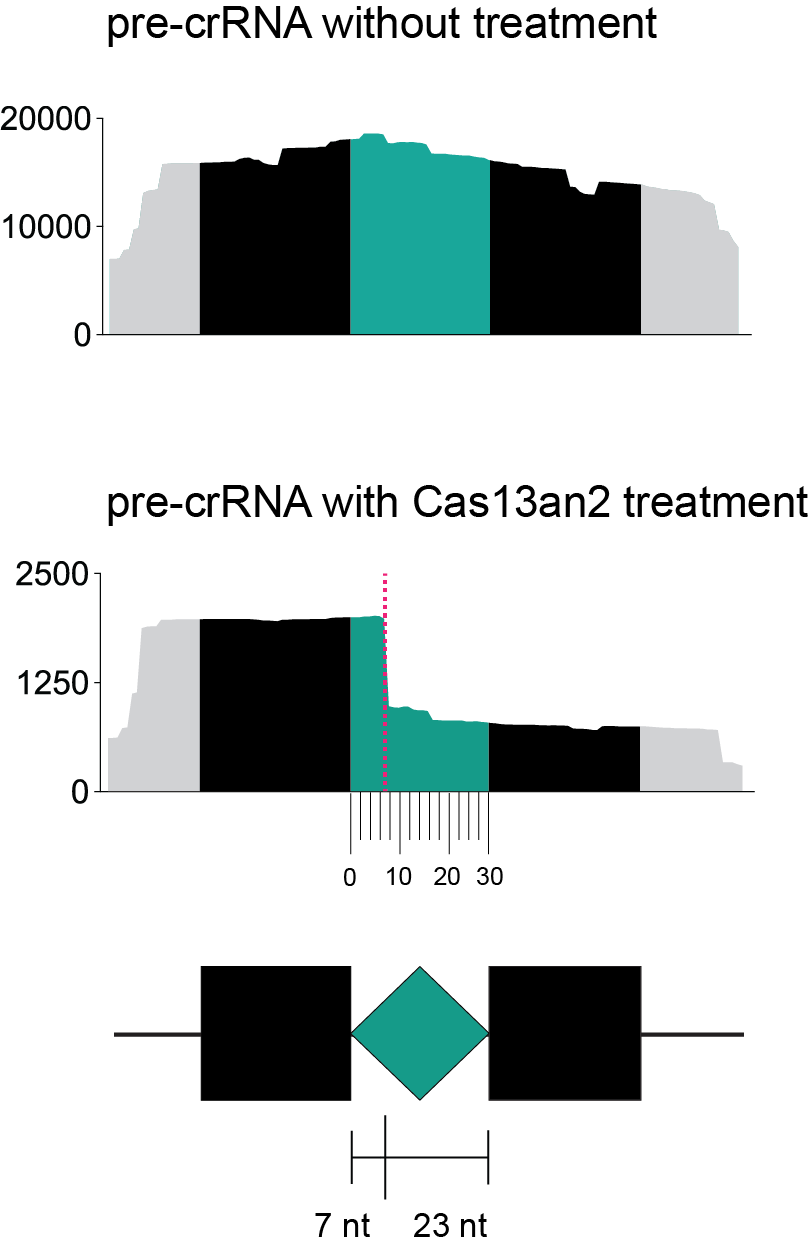
**

**Fig. S12. RNA-sequencing of *in vitro* processed pre-crRNA**

RNA-sequencing reads of *in vitro* processed pre-crRNA mapped to the *in vitro* transcription template shows cutting of pre-crRNA at position 7 of the spacer. Black square represents CRISPR repeat, and green diamond represents spacers.

**Figure S13**
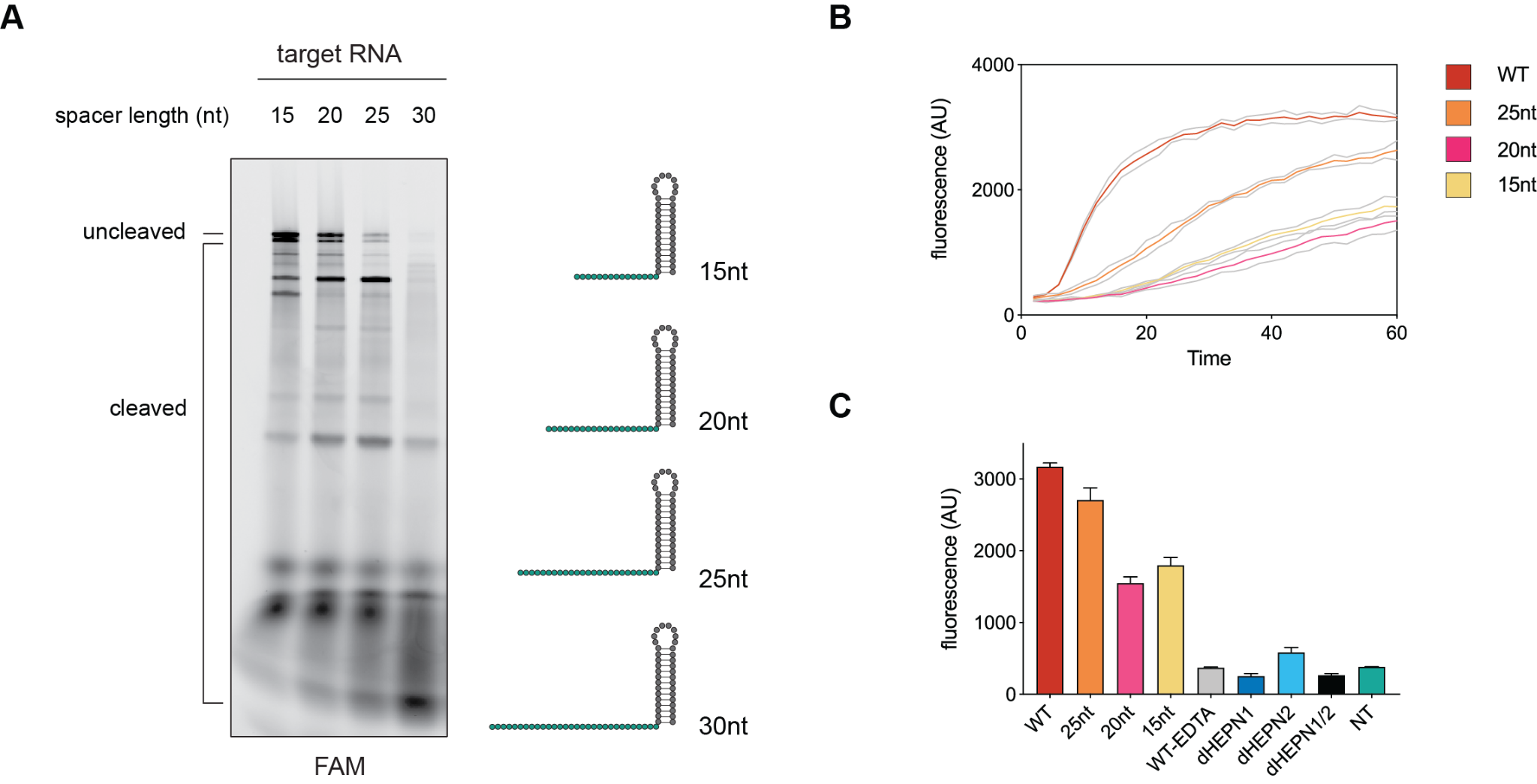


**Fig. S13. Cleavage assay of Cas13an with crRNAs of different spacer lengths.
(A)** 15% denaturing urea-PAGE gel showing target RNA cleavage (*cis-*cleavage) when using crRNAs with spacers of different nt length, and cartoon depiction of crRNAs used in the cleavage assays. Sequences of crRNAs used are in table S11. **(B)** Fluorophore-quencher assay measure of *trans*-cleavage activity when using crRNAs of different spacer length. AU, arbitrary units. **(C)** End point measure of *trans*-cleavage after one hour.

**Figure S14**

**
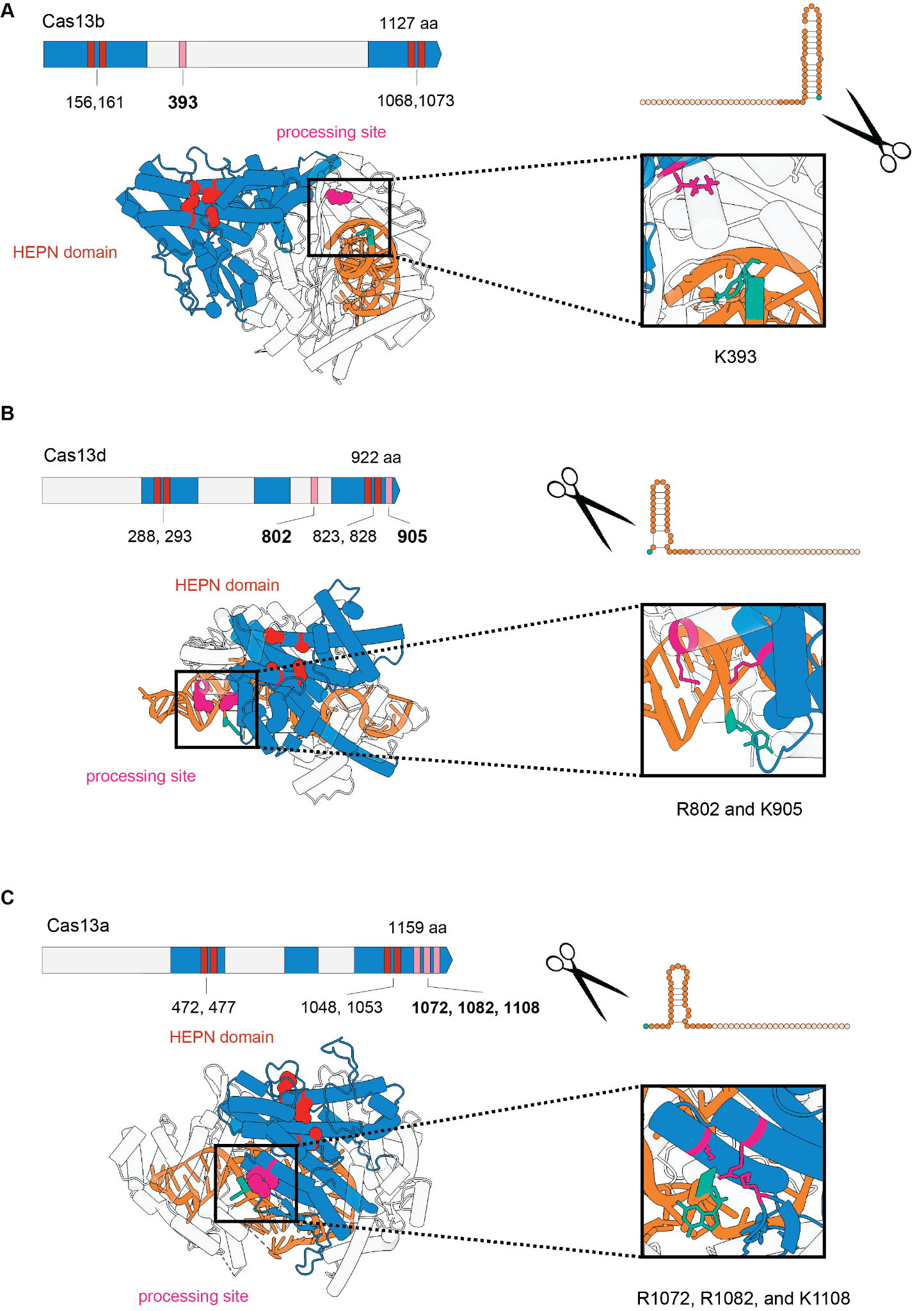
**

**Fig. S14. pre-crRNA processing sites of Cas13**Annotated structures of different Cas13 proteins **(A)** Cas13b (PDBID: 6DTD), **(B)** Cas13d (PDBID: 6IV9), and **(C)** Cas13a (PDBID: 5XWY). Blue indicates HEPN domains, red indicates Rx4H motif of HEPN domains, and magenta indicates residues involved in pre-crRNA processing. Images on right zoom into the secondary guide RNA processing site of different Cas13 subtypes, highlighting catalytic residues in magenta. Shown above the zoomed-in images are cartoon depictions of the crRNA secondary structure and crRNA processing position. Colors on the crRNA mark the following: beige is spacer, orange is CRISPR-repeat, and green is the processing position.

**Figure S15**

**
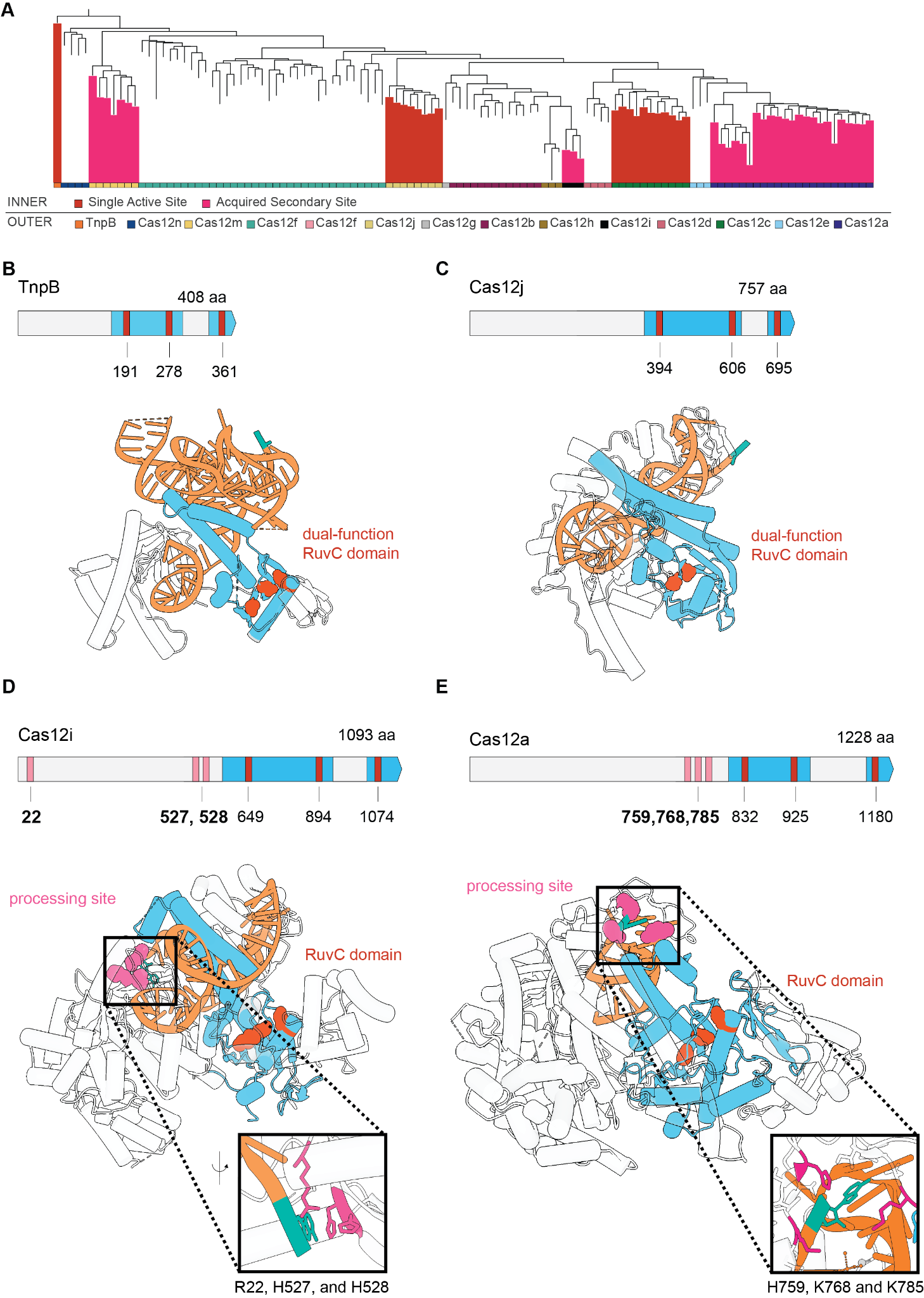
**

**Fig. S15. Recurrent evolution of Cas12 pre-crRNA processing sites
(A)** Phylogenetic tree of TnpB and Cas12 proteins. Red highlights clades that only use a single active site for both guide RNA processing and target cleavage. Magenta highlights clades that acquired a second orthogonal active site for crRNA processing. **(B-E)** Annotated structures of different TnpB and Cas12 proteins. Blue indicates RuvC nuclease domain, red indicates RuvC catalytic residues, and magenta indicates residues in the secondary active site involved in pre-crRNA processing. (B) Annotated structure of ISDra2 TnpB (PDBID: 8H1J). (C) Annotated structure of Cas12j (PDBID: 7M5O). (D) Annotated structure of Cas12i (PDBID: 6W5C). Inset shows the secondary guide RNA processing site of Cas12i where the catalytic residues R22, H527, and H528 are highlighted. (E) Annotated structure of Cas12a (PDBID: 5ID6). Inset shows secondary guide RNA processing site of Cas12a where the catalytic residues H759, K768, and K785 are highlighted.

**Figure S16**

**
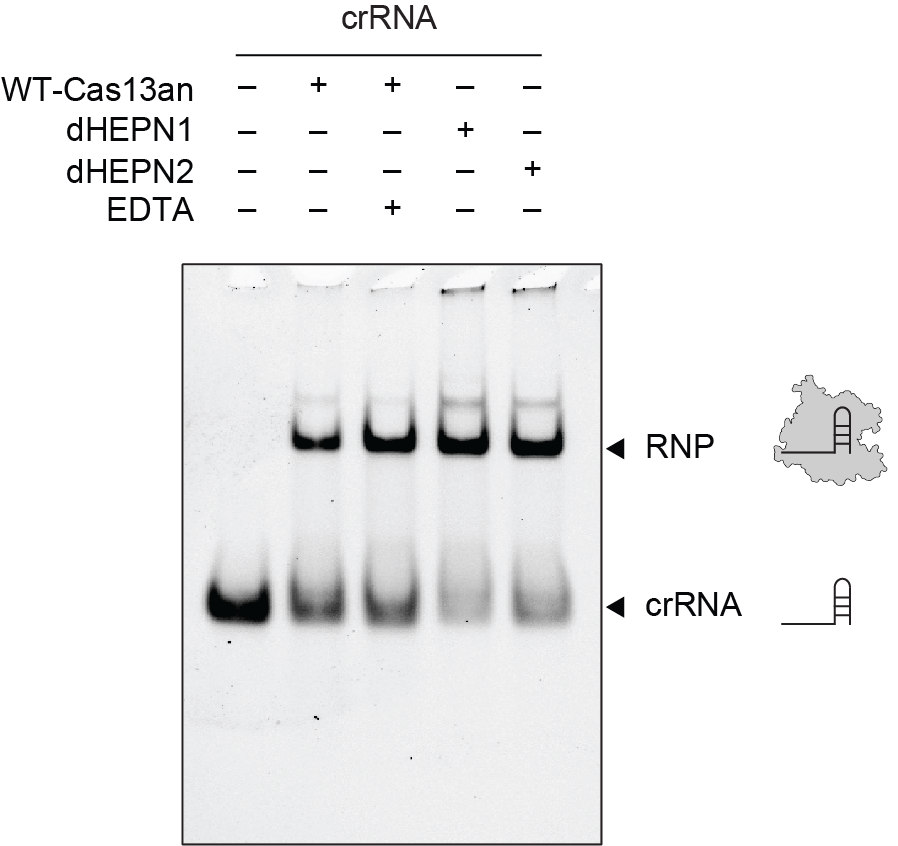
**

**Fig. S16. Electrophoretic mobility shift assay (EMSA) showing ribonucleoprotein complex (RNP) formation in wild-type (WT) Cas13an2 and mutants.**6% native PAGE gel showing Cas13an2 RNP formation, indicated by the upward shifts of crRNA. To note, neither mutations in the HEPN domains nor the addition of EDTA affected RNP formation. Visualized by SYBR-GOLD staining.
